# RAGA prevents tumor immune evasion of LUAD by promoting CD47 lysosome degradation

**DOI:** 10.1038/s42003-023-04581-z

**Published:** 2023-02-23

**Authors:** Lian Zhang, Jing Yu, Mingyue Zheng, Hui Zhen, Qingqiang Xie, Chundong Zhang, Zhongjun Zhou, Guoxiang Jin

**Affiliations:** 1 Medical Research Institute, Guangdong Provincial People’s Hospital (Guangdong Academy of Medical Sciences), Southern Medical University, Guangzhou, 510080 China; 2 School of Biomedical Sciences, Li Ka Shing Faculty of Medicine, University of Hong Kong, Hong Kong, China; 3 College of Basic Medical Sciences, Chongqing Medical University, Chongqing, 400016 China; 4 Department of Surgery Oncology, The Second People’s Hospital of Neijiang, Neijiang, 641000 China; 5 Department of Biochemistry and Molecular Biology, Chongqing Medical University, Chongqing, 400016 China; 6 Guangdong Provincial Geriatrics Institute, Guangdong Provincial People’s Hospital, Guangdong Academy of Medical Sciences, Guangzhou, 510080 China

**Keywords:** Molecular medicine, Cancer

## Abstract

CD47 is a macrophage-specific immune checkpoint protein acting by inhibiting phagocytosis. However, the underlying mechanism maintaining CD47 protein stability in cancer is not clear. Here we show that CD47 undergoes degradation via endocytosis/lysosome pathway. The lysosome protein RAGA interacts with and promotes CD47 lysosome localization and degradation. Disruption of RAGA blocks CD47 degradation, leading to CD47 accumulation, high plasma membrane/intracellular CD47 expression ratio and reduced phagocytic clearance of cancer cells. RAGA deficiency promotes tumor growth due to the accumulation of CD47, which sensitizes the tumor to CD47 blockade. Clinical analysis shows that RAGA and CD47 proteins are negatively correlated in lung adenocarcinoma patient samples. High RAGA protein level is related to longer patient survival. In addition, RAGA^high^CD47^low^ patients show the longest overall survival. Our study thereby not only reveals a mechanism by which RAGA regulates CD47 lysosome degradation, but also suggests RAGA is a potential diagnostic biomarker of lung adenocarcinoma.

## Abstract (web summary)

The lysosome protein RAGA interacts with and promotes lysosome localization and degradation of the macrophage-specific immune checkpoint protein CD47, with possible relevance to lung adenocarcinoma

## Introduction

Cancer tissue composes of cancer cells and microenvironment cells, including immune cells. Cancer progression is therefore not only controlled by cancer cell-intrinsic signaling but also regulated by the immune microenvironment^1,2^. Immunotherapy has led to breakthroughs in revolutionizing targeting therapeutic strategies via evoking the microenvironment immune responses^3–5^. In particular, targeting T cell immune checkpoints with PD-L1/PD1 or CTLA-4 antibodies has shown impressive outcomes in multiple cancers^6–8^. Nevertheless, the efficacy of current strategies blocking T cell immune checkpoints are still not very satisfactory due to low responsive rate or drug resistance. Most of the patients do not benefit well from immunotherapy^9^. Exploring additional regulation mechanisms of immune checkpoints related to not only T cells but also other tumor-associated immune cells may be necessary for developing more effective immunotherapy strategies.

Macrophages, an innate immune cell type, are ubiquitously identified in cancer tissues. Though the activated macrophages could perform phagocytic clearance of cancer cells and antigen presentation to activate adaptive immune responses, the activity is suppressed in most tumor-associated macrophages that even promote tumor growth^10,11^. In parallel to the checkpoints resulting in compromised immune responses of T cells, the CD47-SIRPα axis arises as a macrophage-specific checkpoint^12,13^. CD47 (cluster of differentiation 47), a glycoprotein ubiquitously expressed on the surface of various cancer cells, interacts with the SIRPα protein expressed on macrophages. CD47-SIRPα interaction induces “do not eat me” signal to prevent the phagocytic clearance of cancer cells, thus serving as a potential target for cancer immune therapy in both leukemia and solid tumors^14,15^. Pioneer studies have shown that CD47 can undergo endocytosis trafficking in multiple cellular models. The maintenance of CD47 on the erythroid cell surface is regulated by the actin cytoskeleton. Disruption of actin structure leads to CD47 endocytic trafficking^16^. CD47-SIRPα interaction results in trans-endocytosis of CD47 into adjacent SIRPα expression CHO cells and astrocytes^17^. In addition, ionomycin stimulates the endocytic trafficking of CD47 into the CRACR2a compartment of Jurkat cells^18^. To date, how cancer cell CD47 protein stability is maintained on the cell surface remains to be investigated.

RAGA (RRAGA, Ras-related GTP binding A), a guanine nucleotide-binding protein of either GTP or GDP^19^, has been recognized as an upstream regulator of amino acid-dependent mTORC1 activation. RAGA locates on the lysosome surface, where amino acid stimulation induces the binding of RAGA with GTP, thereby recruiting mTORC1 from the cytoplasm to the lysosome surface for activation^20,21^. Our previous study further reveals that the ubiquitination of RAGA plays a negative feedback role to prevent mTORC1 hyperactivation^22^. Various studies have shown that deregulated mTORC1 signaling is related to diverse human diseases including cancer. mTORC1 inhibition by either inhibitor or knocking down RAPTOR prevents cell growth and cancer progression^23,24^. GATOR1 complex displays GAP activity towards RAGA via RAGA GTP hydrolysis then suppressing amino acid-dependent mTORC1 signaling. The inactivating mutations of GATOR1 correlating with hyperactive mTORC1 were found in certain cancer cells^25^. However, the exact role of RAGA in cancer has not been well characterized.

In addition to activate amino acid-dependent mTORC1, emerging evidences have demonstrated that RAGA can also function independently of mTORC1. Some studies have demonstrated that RAGA may play functions other than mTORC1 through various interaction proteins such as nucleolar protein NG132^26^, microtubule cargo adapter DYLNT^27^, and hedgehog signaling protein WDR35^28^. Interestingly, it has been reported that RAGA expresses in microglia, the resident macrophage in the central nervous system, and is essential for the cell development and function of microglia. RAGA but not mTOR mutants in zebrafish leads to defective lysosome function and microglia phagocytotic flux^29^. However, whether cancer cell RAGA can perform effects on microenvironment macrophage thus modulating cancer immunity remains unknown.

In this study, we demonstrate that CD47 localizes both on the cancer cell surface and in the cytoplasm, particularly colocalizes with endosomes and lysosomes. We reveal that CD47 undergoes degradation via the endocytosis/lysosome pathway. RAGA plays a tumor suppressor role in lung adenocarcinoma by promoting the lysosome degradation of CD47. RAGA knockdown increases CD47 protein level, thereby prevents the engulfing of cancer cells by macrophages and promotes tumor growth. Moreover, the protein level of RAGA is negatively correlated with CD47 and associated with longer survival in clinical lung adenocarcinoma patients. Our study reveals that RAGA serves as the upstream promoting regulator of CD47 degradation and may represent a biomarker for cancer diagnosis.

## Results

### RAGA regulates lung adenocarcinoma progression and macrophage phagocytosis

**Fig. 1:**
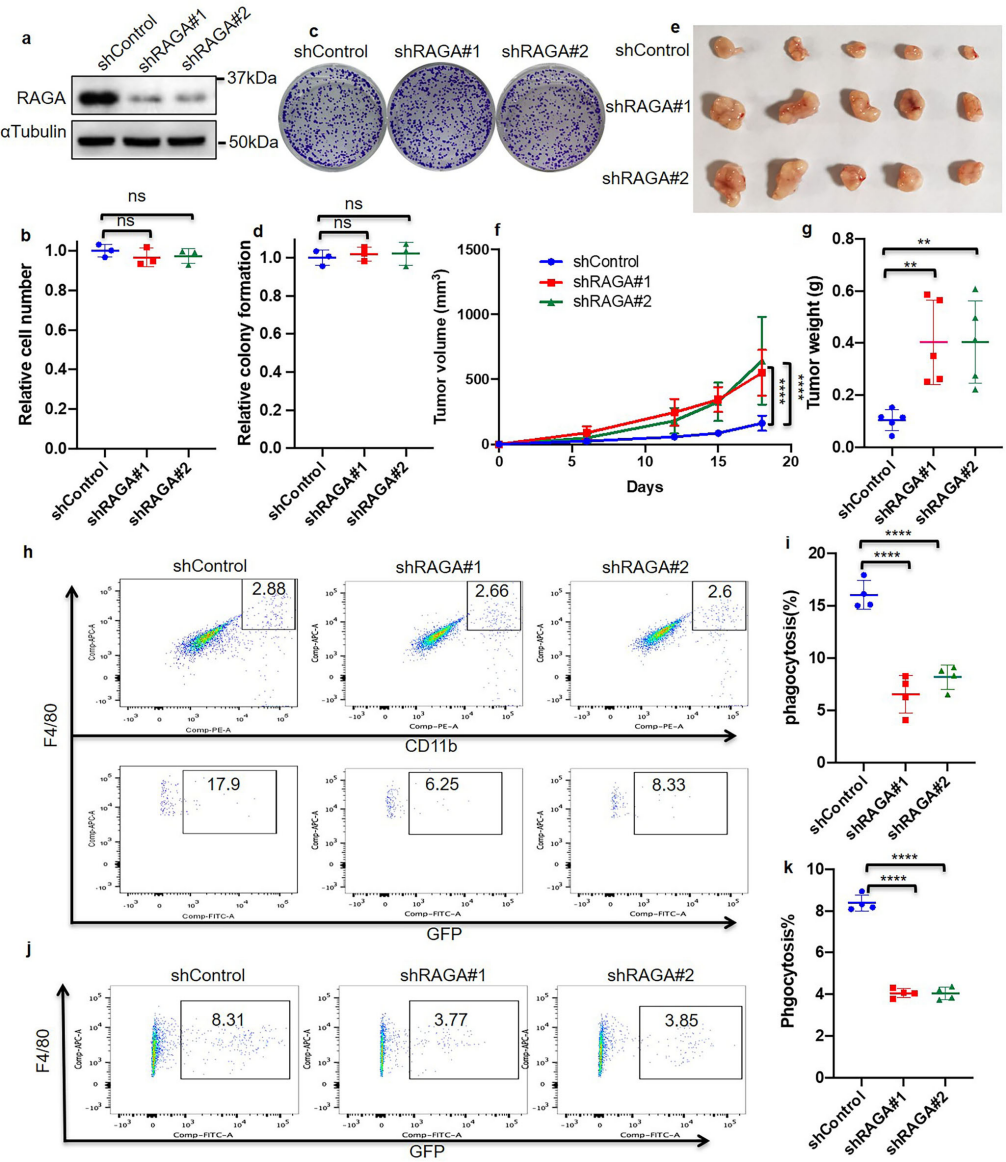
RAGA regulates lung adenocarcinoma progression and macrophage phagocytosis. **a** Immunoblot analysis of indicated proteins in control and RAGA knockdown A549 cells. **b** Relative cell number of control and RAGA knockdown A549 cells (*n* = 3 per group). **c** Representative images of control and RAGA knockdown A549 colonies. **d** Relative colony number of control and RAGA knockdown A549 cells (*n* = 3 per group). **e** The tumor xenografts of control and RAGA knockdown A549 cells. **f** The growth curve of control and RAGA knockdown A549 tumor xenografts (*n* = 5 per group). **g** The weight of control and RAGA knockdown A549 tumor xenografts (*n* = 5 per group). **h** Representative flow cytometry analysis of in vivo phagocytosis in tumor xenografts. Numbers in the upper panel outlined areas indicate the percentage of tumor macrophages. Numbers in the lower panel outlined areas indicate the percentage of tumor macrophages with phagocytosis. **i** Statistical analysis of the percentage of macrophages with phagocytosis in control and RAGA knockdown A549 tumor xenografts (*n* = 4 per group). **j** Representative flow cytometry analysis of in vitro phagocytosis in macrophage and GFP-labeled A549 coculture system. Numbers in the outlined areas indicate the percentage of macrophages with phagocytosis. **k** Statistical analysis of the percentage of macrophages with phagocytosis in the coculture system as in (**j**) (*n* = 4 per group). Data were from two independent experiments. Statistical data were presented as mean ± SD. *P* < 0.05 is considered statistical significance. ** indicates *P* < 0.01; **** indicates *P* < 0.0001; ns indicates not significant.

We performed experiments to explore the role of RAGA in lung adenocarcinoma by in vitro cell culture and in vivo xenograft system. To this end, we generated RAGA knockdown A549 and H1299 cancer cells. In vitro cell proliferation assays showed that RAGA knockdown could not influence both of cell number and colony formation in A549 and H1299 cells (Fig. 1a–d and Supplementary Fig. 1a–d). We further established RAGA control and knockdown A549 tumor xenografts in female Balb/c nude mice. Surprisingly, RAGA knockdown significantly promoted the growth of xenograft tumors. The RAGA knockdown tumors were larger and weighed higher compared to that of control tumors (Fig. 1e–g). The results collectively demonstrate that RAGA depletion promotes the tumor growth of lung adenocarcinoma in vivo but not in vitro.

It is well known that the compromised immune microenvironment contributes to cancer growth^3,4^. We suspected that RAGA knockdown in cancer cells might promote tumor growth via immune suppression by modulating the communication of tumor cells and microenvironment immune cells. As T cells are depleted while macrophages are intact in the Balb/c nude mice, we examined whether macrophage-dependent phagocytosis was regulated by RAGA in tumors.

The RAGA control and knockdown cells we used for the aforementioned cell and xenograft analysis expressed GFP. To examine phagocytosis in tumor xenografts, the fresh tumor tissues were then digested and the isolated cells were stained with CD11b and F4/80 antibodies to label macrophages. Flow cytometry analysis revealed that the macrophage infiltration remained no difference (Supplementary Fig. 1e). However, the percentage of macrophages with engulfed tumor cells, as represented by GFP, CD11b, and F4/80 triple-positive cells (GFP^+^CD11b^+^F4/80^+^), in the total CD11b^+^F4/80^+^ macrophages was significantly lower in the RRAGA knockdown tumor (Fig. 1h, i), supporting that the knockdown of RAGA inhibits tumor macrophage phagocytosis in vivo. We also performed in vitro coculture of mouse bone marrow-derived macrophage cells with GFP-labeled RAGA control and knockdown cancer cells. Flow cytometry analysis demonstrated that the phagocytic activity of in vitro cultured macrophages, as represented by the percentage of GFP^+^F4/80^+^ macrophages in total macrophages, was suppressed when they were cocultured with RAGA knockdown cells compared to that of control cells (Fig. 1j, k). We, therefore, demonstrate that RAGA knockdown in lung adenocarcinoma cells inhibits their phagocytic clearance by macrophages both in vivo and in in vitro coculture systems.

RAGA is a high homology with RAGB. We were wondering whether RAGB has the same functions. However, RAGB was very low or nearly undetectable in A549, H1299 lung carcinoma cells, MDA-MB-231 breast cancer cells, and HEK293T cells (Supplementary Fig. 1f). Previous study has also demonstrated that RAGB express much lower than RAGA^30^. We, therefore, focused our study on the role of RAGA in the regulation of CD47 expression.

### RAGA depletion inhibits phagocytosis and promotes tumor growth via CD47 accumulation

**Fig. 2:**
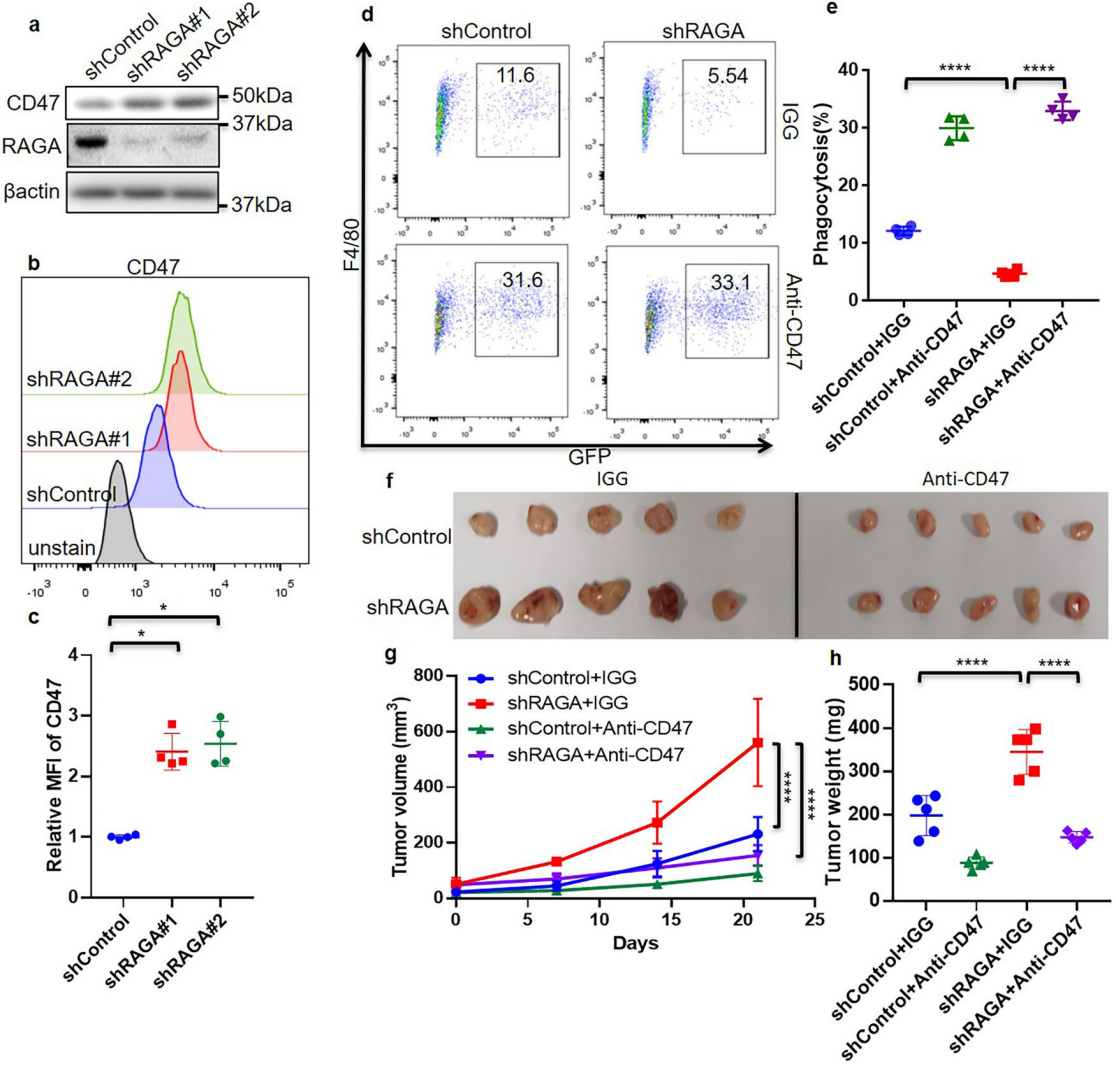
RAGA depletion suppresses phagocytosis and promotes tumor growth via CD47 accumulation. **a** Immunoblot analysis of indicated proteins in control and RAGA knockdown A549 cells. **b** Representative flow cytometry of CD47 protein expression on the cell surface. **c** Statistical analysis of cell surface CD47 expression (*n* = 4 per group). **d** Representative flow cytometry analysis of in vitro phagocytosis in macrophage and GFP-labeled A549 coculture system. Numbers in the outlined areas indicate the percentage of macrophages with phagocytosis. **e** Statistical analysis of the percentage of phagocytic macrophages cocultured with control and RAGA knockdown A549 cells under IGG or anti-CD47 antibody treatment (*n* = 4 per group). **f** The tumor xenografts of control and RAGA knockdown A549 with IGG or anti-CD47 antibody treatment. **g** The growth curve of A549 tumor xenografts is shown in (**f**) (*n* = 5 per group). **h** The weight of A549 tumor xenografts is shown in (**f**) (*n* = 5 per group). Data were from two or three independent experiments. Statistical data were presented as mean ± SD. *P* < 0.05 is considered statistical significance. *indicates *P* < 0.05 and **** indicates *P* < 0.0001.

CD47, a transmembrane protein expressed on the cancer cell surface, is a critical immune checkpoint via interacting with macrophage surface protein SIRPα to prevent phagocytosis^12,13^. CD47 antibodies have been utilized to block CD47 and promote phagocytic clearance of tumor cells in various preclinical studies. Clinical trials of CD47 blockade with anti-CD47 monoclonal antibodies also show potential efficacy in treating various cancer types^31–33^. We have shown that RAGA knockdown inhibits cancer cell phagocytosis by macrophages. We, therefore, sought to determine whether RAGA is an upstream suppressor of CD47. In support of this notion, the knockdown of RAGA enhanced the CD47 protein levels in multiple cancer cells, but not the mRNA level (Fig. 2a and Supplementary Fig. 2a–d). The flow cytometry approach further revealed the increased accumulation of cell surface CD47 (Fig. 2b, c).

We next sought to determine whether RAGA knockdown inhibits phagocytosis indeed through increasing CD47 protein accumulation. To this end, we blocked CD47 by treating the control and RAGA knockdown A549 cells with anti-CD47 monoclonal antibody (B6H12) and examined cell phagocytosis by cocultured macrophages. Consistent with the result mentioned above (Fig. 1j, k), RAGA knockdown inhibited phagocytosis (Fig. 2d, e). CD47 antibody promoted the phagocytosis of both control and knockdown tumor cells by macrophages (Fig. 2d, e). Notably, the CD47 antibody dramatically restored the phagocytosis of RAGA knockdown cells to the level of control cells (Fig. 2d, e), supporting the notion that the phagocytic inhibition of RAGA knockdown tumor cells is dependent on increased CD47 accumulation.

To further determine whether CD47-mediated phagocytic inhibition is responsible for the elevated tumor growth upon RAGA knockdown, a monoclonal antibody was used to inhibit CD47 in the in vivo xenograft experiment. CD47 blockade suppressed the growth of control xenografts. On the other hand, though RAGA knockdown dramatically increased the tumor growth compared to that of control, the promoting effect was repressed by CD47 blockade (Fig. 2f–h), supporting that the oncogenic effect of RAGA knockdown is largely due to upregulated CD47 accumulation.

### RAGA regulates CD47 and phagocytosis independent of the conversion of RAGA GTP/GDP binding states

**Fig. 3:**
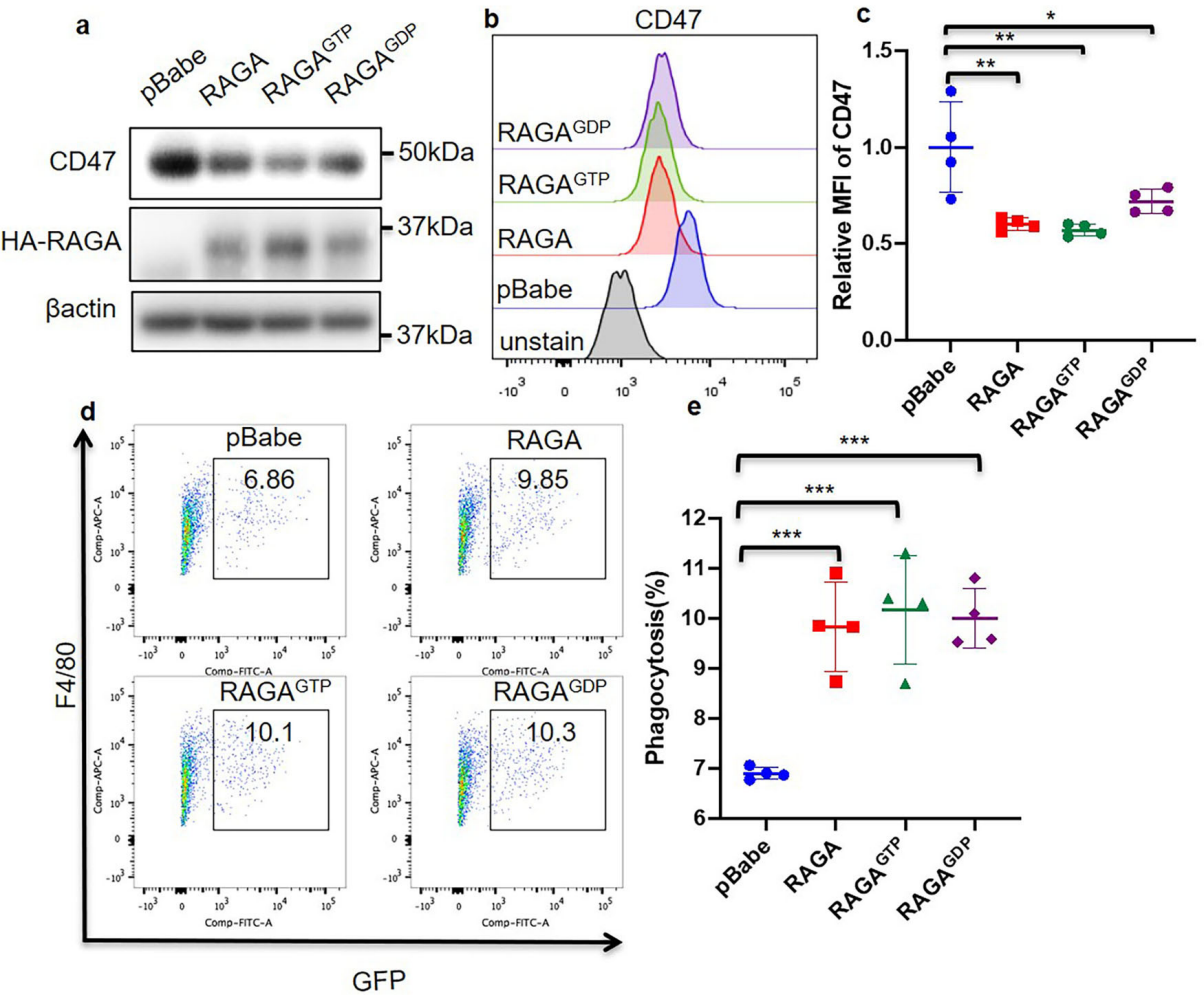
Both RAGA GTP/GDP binding states inhibit CD47 and promote phagocytosis. **a** Immunoblot analysis of indicated proteins in pBabe empty vector, wild type or mutant RAGA overexpressed A549 cells. **b** Representative flow cytometry of CD47 protein expression on the cell surface. **c** Statistical analysis of cell surface CD47 expression (*n* = 4 per group). **d** Representative flow cytometry analysis of in vitro phagocytosis in macrophage and GFP-labeled A549 coculture system. Numbers in the outlined areas indicate the percentage of macrophages with phagocytosis. **e** Statistical analysis of the percentage of phagocytic macrophages cocultured with empty vector, wild type or mutant RAGA overexpressed A549 as in (**d**) (*n* = 4 per group). Data were from two or three independent experiments. Statistical data were presented as mean ± SD. *P* < 0.05 is considered statistical significance. * indicates *P* < 0.05; **indicates *P* < 0.01; and *** indicates *P* < 0.001.

In contrast to CD47 accumulation upon RAGA knockdown, RAGA overexpression reduced CD47 total and surface expression levels (Fig. 3a–c and Supplementary Fig. 3a). It is known that RAGA is a GTP or GDP binding protein. Amino acids stimulate the conversion of the RAGA GDP binding state to the GTP binding state, which is an essential step for the activation of mTORC1 signaling on the lysosome surface^20,21^. We, therefore, examined whether the inhibition of CD47 expression is dependent on amino acid stimulation and RAGA GTP/GDP binding states. We found that amino acid stimulation did not influence CD47 protein expression (Supplementary Fig. 3b, c). We also generated constitutive GTP or GDP binding RAGA mutants RAGA^GTP^(RAGA^Q66L^) and RAGA^GDP^(RAGA^T21L^). Notably, both RAGA^GTP^ and RAGA^GDP^ inhibited CD47 total and cell surface expression levels similar to that of wild-type RAGA (Fig. 3a–c). Amino acid-dependent conversion between GTP and GDP binding states of RAGA is therefore not required for inhibiting CD47 expression, indicating that RAGA reduces CD47 protein level in an mTORC1-independent manner.

We then investigated the role of GTP/GDP binding states on phagocytosis. To this end, we cocultured mouse bone marrow-derived macrophages with GFP-labeled A549 cells overexpressing RAGA, RAGA^GTP^, RAGA^GDP^, or control vector. Flow cytometry analysis was used to analyze the phagocytosis activity, as represented by the percentage of GFP and F4/80 double-positive macrophages in total macrophages. In contrast to the result found in RAGA knockdown cells, RAGA overexpression promoted phagocytosis and RAGA^GTP^, RAGA^GDP^ showed the same promotion efficacy as wild-type RAGA (Fig. 3d, e). Therefore, RAGA regulates phagocytosis independent of the conversion of RAGA GTP/GDP binding states.

### RAGA promotes CD47 lysosome degradation

**Fig. 4:**
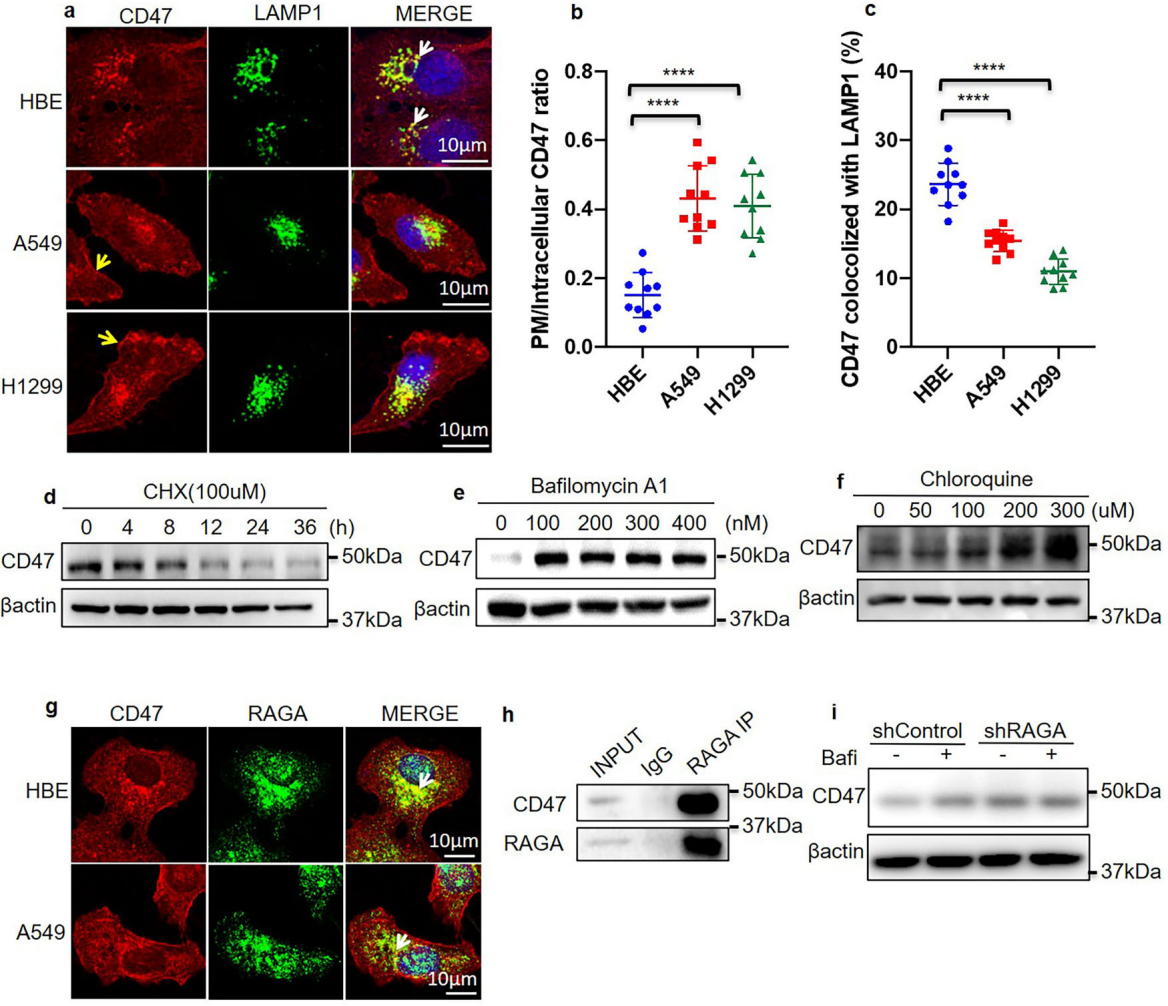
RAGA promotes CD47 lysosome degradation. **a** Immunofluorescence staining of CD47 and LAMP1 in HBE, A549, and H1299 cells. The yellow arrow indicates cell surface CD47 staining. The white arrow indicates the colocalization of CD47 with LAMP1. **b** Statistical analysis of PM/intracellular CD47 expression ratio (*n* = 10 cells per group). **c** Statistical analysis of the percentage of CD47 colocalized with LAMP1 (*n* = 10 image areas per group). **d** Immunoblot analysis of the indicated proteins in 100 μM cycloheximide (CHX) treated A549 cells. h indicates hours. **e** Immunoblot analysis of the indicated proteins in A549 cells treated with different dosages of bafilomycin A1. **f** Immunoblot analysis of the indicated proteins in A549 cells treated with different dosages of chloroquine. **g** Immunofluorescence staining of CD47 and RAGA in HBE and A549 cells. The white arrow indicates the colocalization of CD47 with RAGA. **h** Immunoblot analysis of indicated proteins assessed after RAGA immunoprecipitation. **i** Immunoblot analysis of indicated proteins in control and RAGA knockdown A549 cells treated with or without bafilomycin A1. Bafi indicates bafilomycin A1. Data were from two independent experiments. Statistical data were presented as mean ± SD. *P* < 0.05 is considered statistical significance. **** indicates *P* < 0.0001.

Localization of CD47 on the cancer cell surface is essential for preventing phagocytic clearance. We stained CD47 in a clinical lung adenocarcinoma tissue sample with an immunohistochemistry approach. Interestingly, we noticed that CD47 was not only localized on the peripheral cell plasma membrane but also in the intracellular cytoplasm (Supplementary Fig. 4a). We further analyzed CD47 expression and localization in the cultured A549, H1299 lung carcinoma cells, and HBE normal human bronchial epithelial cells. The protein level of CD47 was higher in A549, H1299 cancer cells than in HBE normal cells (Supplementary Fig. 4b). Similar to that observed in the patient samples, both plasma membrane and intracellular CD47 localization were identified in all of the cultured cells (Fig. 4a). By measuring the intensity of peripheral and intracellular CD47, the ratio of CD47 on the plasma membrane to that in the cytoplasm (PM/intracellular CD47 ratio) was elevated in both A549 and H1299 lung cancer cells in comparison to HBE normal epithelial cells (Fig. 4b), suggesting that cancer cells favor to preserve the membrane localization of CD47.

How CD47 protein stability is maintained on cancer cell plasma membrane remains to be further answered. Membrane proteins could be internalized and then transported to the lysosome for degradation^34^. Interestingly, we found CD47 partially colocalized with LAMP1, a lysosome marker, in all of HBE normal epithelial cells and A549, H1299 cancer cells. Moreover, the percentage of CD47 colocalized with LAMP1 decreased in A549, H1299 cancer cells compared to that in HBE normal cells (Fig. 4a, c). These data indicates that CD47 might be relocalized to the lysosome for degradation.

It is currently poorly understood about the mechanisms involved in the regulation of CD47 protein degradation in cancer. We treated A549 cells with cycloheximide(CHX), which blocks protein translation, and found that the CD47 protein level gradually decreased upon CHX treatment (Fig. 4d), supporting the notion that the protein degradation pathway was involved in the regulation of CD47 protein homeostasis. The degradation of cellular proteins is usually via proteasome and/or lysosome pathway^35,36^. To determine which pathway is responsible for the degradation of CD47, we treated the cells with MG132 or bafilomycin A1 to inhibit proteasome- and lysosome-mediated protein degradation respectively. Notably, bafilomycin A1, the lysosome inhibitor, dramatically increased the accumulation of CD47 (Fig. 4e and Supplementary Fig. 4c), while MG132, the proteasome inhibitor, might slightly increase CD47 protein level (Supplementary Fig. 4d). Another lysosome inhibitor chloroquine also caused the accumulation of CD47 in both A549 and H1299 cells (Fig. 4f and Supplementary Fig. 4e). These data demonstrate that CD47 undergoes degradation in a lysosome-dependent manner.

We have shown that the lysosome protein RAGA is an upstream regulator of CD47 protein level and CD47 undergoes lysosome-dependent degradation. Furthermore, by performing an immunofluorescence assay, we found that RAGA partially colocalized with CD47 in both HBE normal cells and A549 lung cancer cells (Fig. 4g). Moreover, coimmunoprecipitation assay showed that endogenous RAGA interacted with endogenous CD47 (Fig. 4h). We performed the co-ip of RAGA with lysosome protein LAMP2 in the experiment with the same condition and confirmed that RAGA interacted with CD47 but not LAMP2 (Supplementary Fig. 4f). These evidences raise the possibility that RAGA inhibits CD47 expression by recruiting it to lysosome for degradation.

To confirm the role of RAGA in CD47 lysosome degradation, we treated control and RAGA knockdown A549 and H1299 cells with bafilomycin A1 respectively. Both RAGA knockdown and bafilomycin treatment alone caused the accumulation of CD47 compared to the control cells without RAGA knockdown and bafilomycin treatment. However, bafilomycin no longer further enhanced CD47 expression in RAGA knockdown cells (Fig. 4i and Supplementary Fig. 4g), verifying that the accumulation of CD47 in RAGA knockdown cells is due to a repression of lysosome degradation.

### RAGA promotes the localization of CD47 to lysosome

We then sought to investigate how RAGA promotes CD47 lysosome degradation. One of the classical lysosome degradation pathways is through endocytosis. The membrane protein is internalized into the endosome and then the late-stage endosome is fused with a lysosome to drive degradation. To determine whether CD47 undergoes endocytosis, the internalization inhibitor dynasore was used to treat the cells, and increased CD47 protein level and its membrane accumulation were confirmed (Supplementary Fig. 5a, b). Simultaneously, the amount of lysosome CD47 was decreased (Supplementary Fig. 5c), supporting that plasma membrane CD47 undergoes endocytosis and following lysosome degradation.

**Fig. 5:**
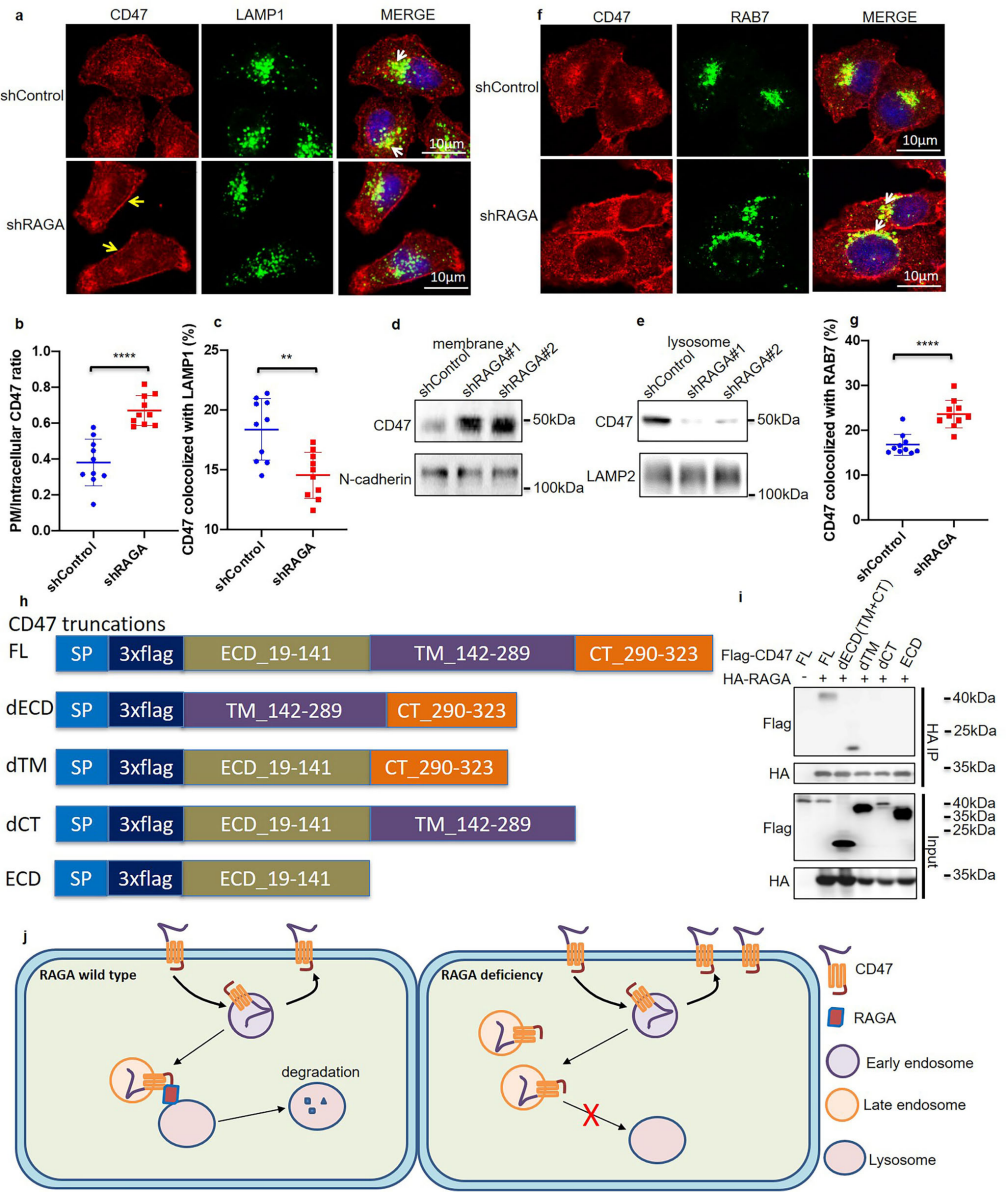
RAGA promotes CD47 lysosome localization. **a** Immunofluorescence staining of CD47 and LAMP1 in control and RAGA knockdown A549 cells. The yellow arrow indicates cell surface CD47 staining. The white arrow indicates the colocalization of CD47 with LAMP1. **b** Statistical analysis of PM/intracellular CD47 expression ratio (*n* = 10 cells per group). **c** Statistical analysis of the percentage of CD47 colocalized with LAMP1 (*n* = 10 image areas per group). **d** Immunoblot analysis of indicated proteins in the isolated plasma membrane fraction of control and RAGA knockdown cells. **e** Immunoblot analysis of indicated proteins in the isolated lysosome fraction of control and RAGA knockdown cells. **f** Immunofluorescence staining of CD47 and RAB7 in control and RAGA knockdown A549 cells. The white arrow indicates the colocalization of CD47 with RAB7. **g** Statistical analysis of the percentage of CD47 colocalized with RAB7 (*n* = 10 image areas per group). **h** Construction of CD47 truncation mutants. **i** Immunoprecipitation analysis of the interaction between RAGA and CD47 truncation mutants. **j** Schematic model. RAGA interacts with CD47 TM and CT domains to promote the fusion of CD47-specific late endosomes with lysosomes, driving CD47 lysosome degradation. Loss of RAGA prevents the fusion of CD47 endosomes with lysosomes that causes the accumulation of late endosomes and increased CD47 levels. Data were from two independent experiments. Statistical data were presented as mean ± SD. *P* < 0.05 is considered statistical significance. ** indicates *P* < 0.01 and **** indicates *P* < 0.0001.

We then performed an immunofluorescence analysis of CD47 in control and RAGA knockdown cells. Knockdown of RAGA not only enhanced the expression of CD47, but also increased the ratio of PM/intracellular CD47 levels in cancer cells (Fig. 5a, b). In addition, the percentage of CD47 colocalized with lysosome marker LAMP1 decreased upon RAGA depletion (Fig. 5a, c). Consistently, fraction isolation and immunoblot assay also showed that the amount of CD47 protein was accumulated on the plasma membrane, but decreased in the lysosome of RAGA knockdown cells (Fig. 5d, e). On the contrary, the amount of membrane-localized CD47 reduced, whereas lysosome located CD47 elevated upon RAGA overexpression (Supplementary Fig. 5d, e), suggesting that RAGA drives the relocalization of CD47 from membrane to lysosome.

A key step of the endolysosome degradation pathway is the fusion of RAB7 positive late endosome with the lysosome, thereby transporting the protein substrate to the lysosome for the destiny of degradation^37,38^. As RAGA locates on the lysosome surface, we thought RAGA might be required for the transport of CD47 from the endosome to the lysosome. To test this hypothesis, we examined the colocalization of CD47 with late endosome marker RAB7. The percentage of CD47 colocalized with RAB7 was significantly enhanced upon the disruption of RAGA expression (Fig. 5f, g). Together with the reduced localization of CD47 on lysosome (Fig. 5a, c), our data suggest that RAGA knockdown prevents the fusion of lysosome with CD47 containing late endosome, thereby inhibiting CD47 transportation from endosome to lysosome for degradation

CD47 protein consists of an N-terminal extracellular domain (ECD), a multiple-spanning transmembrane domain (TM), and a short C-terminal cytosolic tail domain (CT). The endosomes specific for CD47 should present CD47 cytosolic TM domain and CT domain on the surface. To determine the binding domains of CD47 with RAGA, we constructed multiple 3xflag-tagged CD47 truncation mutants by deleting ECD (dECD, containing both TM and CT domains), deleting TM (dTM) or deleting CT (dCT) and we also generated the mutant containing ECD only (Fig. 5h). We then performed the coimmunoprecipitation assay and found that RAGA interacted with the full length (FL) CD47 and dECD mutant, but not ECD mutant. In addition, either dTM or dCT abolished the interaction with RAGA (Fig. 5i). Our data demonstrate both TM and CT domains of CD47 are needed for RAGA interaction. The interaction of RAGA with CD47 TM and CT domain may promote the fusion of lysosomes with CD47-specific late endosomes, leading to CD47 lysosome degradation. Knockdown of RAGA prevents the interaction of CD47 late endosomes with RAGA lysosomes, resulting in accumulated late endosomes. The blockade of endosome/lysosome fusion might promote the recycling of CD47 to the cell surface and increase the level of membrane protein (Fig. 5j).

### Clinical relevance of RAGA/CD47 axis

To determine the clinical relevance of the RAGA/CD47 axis in human patients, we first performed the analysis of the GEO lung adenocarcinoma database on the kmplot.com website. The patients were divided into RAGA^low^/RAGA^high^ or CD47^low^/CD47^high^ subgroups by using the median mRNA expression value (50%) as cut-off points. The result showed that a low level of *RAGA* mRNA was significantly related to poor patient survival (Supplementary Fig. 6a). However, a high mRNA level of CD47 was unexpectedly associated with longer but not shorter survival time (Supplementary Fig. 6b), which conflicted with the oncogenic role of CD47. The finding raises the possibility that the protein stability but not only transcriptional regulation of CD47 might be critical for its involvement in cancer progression.

**Fig. 6:**
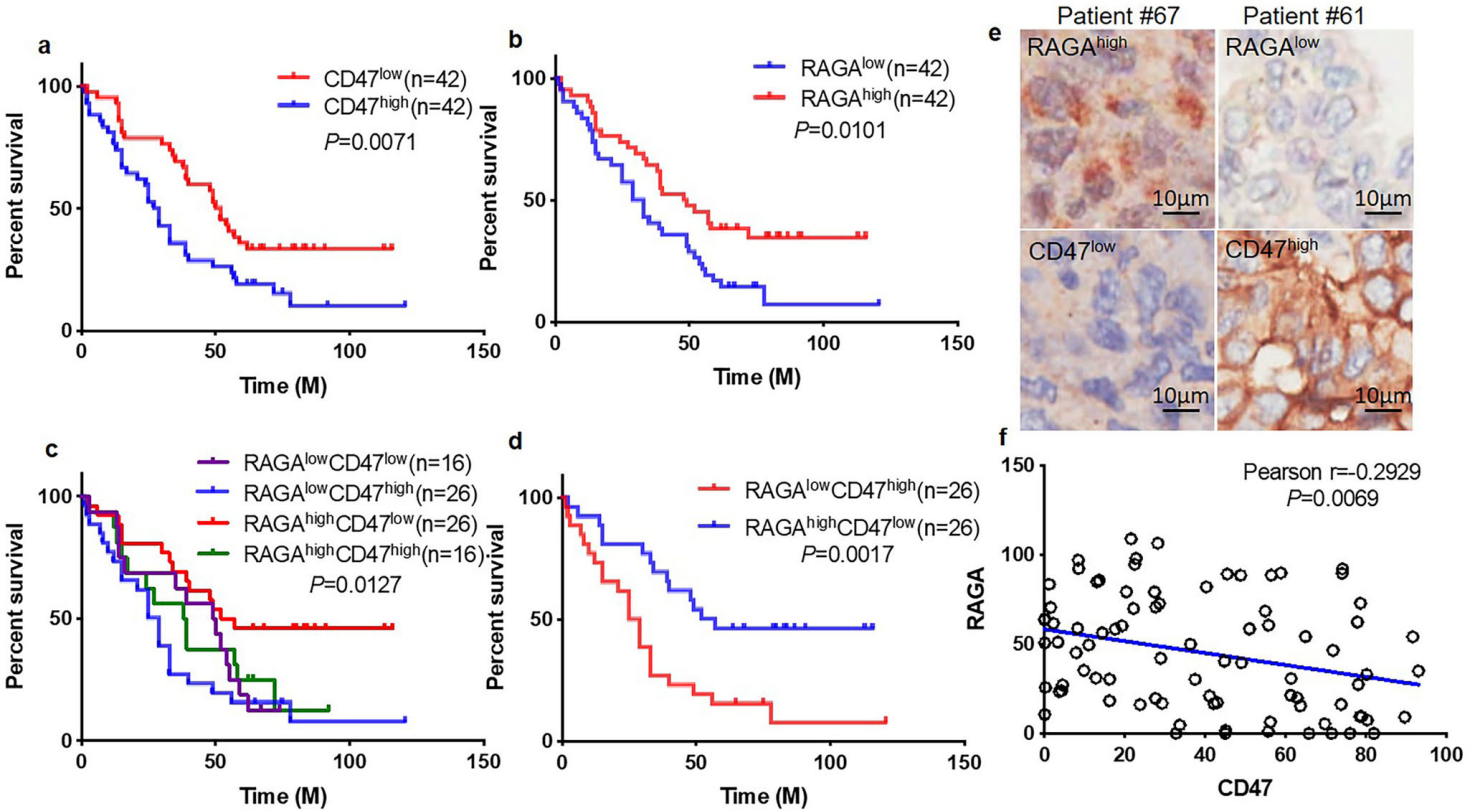
Clinical relevance of RAGA/CD47 axis. **a** Overall survival analysis of 84 lung adenocarcinoma patients with low and high CD47 protein expression. *P* = 0.0071. **b** Overall survival analysis of 84 lung adenocarcinoma patients with low and high RAGA protein expression. *P* = 0.0101. **c** Overall survival analysis of 84 lung adenocarcinoma patients with RAGA^high^CD47^high^, RAGA^high^CD47^low^, RAGA^low^CD47^high^, and RAGA^low^CD47^low^ protein expression. *P* = 0.0127. **d** Overall survival analysis of lung adenocarcinoma patients with RAGA^high^CD47^low^ and RAGA^low^CD47^high^ protein expression. *P* = 0.0017. **e** Representative immunohistochemistry staining of RAGA^high^CD47^low^ and RAGA^low^CD47^high^ patient samples. **f** Pearson correlation coefficient analysis of RAGA and CD47 expression in 84 lung adenocarcinoma patients. Pearson *r* = −0.2929. *P* = 0.0069. *P* < 0.05 is considered statistical significance.

We, therefore, performed a clinical analysis of RAGA and CD47 protein expression in a commercial tissue chip of lung adenocarcinoma patient samples with an immunohistochemistry approach. Eighty-four patients with survival information were used for follow-up analysis (Supplementary Table 1). The patients were divided into subgroups by using RAGA or CD47 median protein expression value as cut-off points. The high level of CD47 protein was expectedly related to poor patient survival (Fig. 6a). On the contrary, the high level of RAGA protein was significantly associated with longer overall survival of patients (Fig. 6b). In addition, the patients were divided into four subgroups according to the median expression of both RAGA and CD47: RAGA^high^CD47^high^(*n* = 16), RAGA^high^CD47^low^(*n* = 26), RAGA^low^CD47^high^(*n* = 26), RAGA^low^CD47^low^(*n* = 16). Among them, RAGA^high^CD47^low^ patients displayed significantly longer survival than other groups of patients (Fig. 6c, d). Moreover, in support of the notion that RAGA promotes CD47 degradation, the RAGA protein level was significantly negatively correlated with the CD47 protein level in the cohort of patients (Fig. 6e, f).

The clinical analysis not only supports the mechanistic role of RAGA in promoting CD47 degradation, but also reveals that RAGA alone and in combination with CD47 might be potential molecular markers in lung adenocarcinoma diagnosis.

## Discussion

Lung cancer has become one of the most malignant cancers, accoutering for the leading cause of cancer-associated mortality worldwide^39,40^. Blockade of T cell immune checkpoints via targeting PD1/PD-L1 and/or CTLA-4 puts forward the development of tumor immunotherapy strategies including lung cancer, but the response rates are still limited^8,41,42^. On the other hand, the interaction of cancer cell CD47 with macrophage SIRPα inhibits phagocytosis to promote tumor progression^5,12^. Targeting the CD47-SIRPα checkpoint is also a potential strategy for lung cancer immunotherapy^43,44^. In this study, we reveal RAGA as a negative regulator of CD47 stability thus involved in the regulation of phagocytosis and lung adenocarcinoma progression.

Maintaining protein stability by preventing its degradation is important for immune checkpoint protein expression in cancer. A large number of studies have demonstrated that the T cell checkpoint protein PD-L1 is degraded through either proteasome- or lysosome- pathways and cancer cell inhibits PD-L1 degradation via PD-L1 glycosylation, palmitoylation, or many other mechanisms^45–48^. As for CD47, multiple transcriptional mechanisms have been reported to promote CD47 expression in cancer. The oncogenic protein MYC binds directly to the promoters of both PD-L1 and CD47 genes and drives their transcription^49^. Hypoxia-inducible factor 1 (HIF-1) elevates CD47 transcription in breast cancer^50^. NFκB is also involved in CD47 transcriptional upregulation in several cancers^51,52^. Interestingly, we found that high *CD47* mRNA expression was associated with longer patient survival in the GEO lung adenocarcinoma database, while on the contrary, the protein level of CD47 predicted poor patient survival in our cohort of patients, suggesting the regulation of CD47 protein stability in cancer is very important. However, how CD47 protein stability or degradation is controlled in cancer is still unclear. In this study, we demonstrate that CD47 can be degraded through the endocytosis/lysosome pathway and the degradation is promoted by the RAGA protein in lung adenocarcinoma.

It is known that GTP-loaded RAGA stimulates amino acid-dependent mTORC1 signaling. The GATOR1 complex promotes RAGA GTP hydrolysis to inhibit mTORC1. A previous report identifies inactivating mutations of GATOR1 components DEPDC5 and NPRL2 in glioblastoma and ovarian cancers^25^, suggesting a potential correlation of RAGA GTP loading with cancer growth. However, though the study provides the correlation of GATOR1 mutation with cancer, whether GATOR1 indeed inhibits tumor growth and whether it is dependent on RAGA or mTORC1 inhibition are not answered. In contrast, another study demonstrates that DEPDC5 knockout displays no significant effect on either cancer cell viability or tumor growth. Moreover, there is a paper showing that GTP binding RagA^GTP/GTP^ constitutive activates mTORC1 during fasting but cannot increase mTORC1 activity in a steady condition of complete culture medium. RagA^GTP/GTP^ cells has a normal proliferation rate. RagA^GTP/GTP^ mouse embryos develop normal^30^. The findings suggest that the role of the GATOR1-RAGA axis in cell proliferation and tumor progression should be carefully evaluated^53^. The findings also support our data that RAGA has no influence on LUAD cell growth. Though RAGA does not affect cell proliferation, we demonstrate that RAGA serves as a tumor suppressor in vivo. RAGA knockdown promoted LUAD tumor progression due to the accumulation of macrophage checkpoint CD47. RAGA overexpression was correlated with longer patient survival in clinical samples.

Despite that RAGA is well recognized to activate mTORC1 by recruiting mTORC1 complex to the lysosome, some studies have demonstrated that RAGA might also play functions other than mTORC1 through various interaction proteins such as nucleolar protein NG132^26^, microtubule cargo adapter DYLNT^27^, and hedgehog signaling protein WDR35^28^. In this study, we identify CD47 as a new RAGA interaction protein and RAGA promotes CD47 lysosome degradation in a mTORC1-independent manner. We rule out the possibility that RAGA regulates CD47 turnover through mTORC1 signaling because of several evidences. Firstly, RAGA must bind GTP to activate mTORC1. In contrast, RAGA GDP binding represses mTORC1^20,21^. Our data showed that both RAGA mutants that constitutively bound to GTP or GDP repressed the total protein and surface expression of CD47 similar to the wild-type RAGA. Secondly, RAGA-mediated mTORC1 activation is dependent on the stimulation of amino acids^20,21^, but we found that the supply of amino acids did not influence the protein expression level of CD47.

Our study collectively demonstrates that CD47 undergoes lysosome-dependent degradation in lung adenocarcinoma. We uncover RAGA as a CD47 negative regulator by promoting its lysosome degradation, thus suppressing tumor growth and serves as a potential diagnostic marker of cancer malignancy.

## Methods

### Cell culture

Human H1299, A549 lung adenocarcinoma cells, HBE normal bronchial epithelial cells, and HEK293T cells were cultured in Dulbecco’s Modified Eagle Medium (DMEM) (Gibco) supplied with 10% fetal bovine serum (FBS) (Hyclone) and 100 U/ml penicillin-streptomycin (Hyclone) in cell incubator with humidified 37 °C temperature and 5% CO_2_. Bafilomycin A1 (Selleck) and chloroquine (Sigma-Aldrich) were used to prevent lysosome-dependent protein degradation and MG132 (Selleck) was used to inhibit proteasome degradation in the cultured cells. Dynasore (Selleck) and cycloheximide(CHX) (Selleck) were added into the culture medium to inhibit membrane internalization and protein synthesis respectively.

### Quantitative real-time PCR (qRT-PCR)

mRNA expression level of *CD47* was analyzed by qRT-PCR. Briefly, the total RNA was extracted from control and RAGA knockdown cells using the RNA fast200 kit (Fastagen). The RNA was converted into cDNA according to the manufacturer’s protocol of the SuperScript III reverse transcriptase kit (Life Technologies). qRT-PCR was then performed by using the following primers:

CD47 forward primer: AGAAGGTGAAACGATCATCGAGC;

CD47 reverse primer: CTCATCCATACCACCGGATCT.

GAPDH forward primer: ACAACTTTGGTATCGTGGAAGG;

GAPDH reverse primer: GCCATCACGCCACAGTTTC.

### Immunoblot and immunoprecipitation

For immunoblot analysis, the cells were lysed in RIPA lysis buffer (50 mM Tris-HCL, 150 mM NaCl, 1%NP40, 0.5% Na deoxycholate, and 1 mM EDTA) containing protease inhibitor cocktail (Roche) and phosphatase inhibitors (Beyotime). The total protein was extracted and followed by SDS-PAGE gel electrophoresis to analyze the expression of indicated proteins. For immunoprecipitation analysis, the cells were lysed in RIPA lysis buffer containing a protease inhibitor cocktail (Roche) and phosphatase inhibitors (Beyotime). The cell lysates were incubated with primary antibodies overnight at 4 °C. Protein agarose A/G beads were then added for an additional 3 h. The beads were washed four times with RIPA lysis buffer and analyzed by immunoblot. The primary antibodies including anti-RAGA(D8B5) rabbit mAb (CST, 4357; dilution 1:1000), anti-CD47 rabbit mAb (Abcam, ab175388; dilution 1:1000), anti-CD47 mouse mAb (Abcam, ab3283; dilution 1:1000), anti-HA mouse mAb (Sigma, H9658; dilution 1:3000), anti-flag mouse mAb (Sigma, F1804; dilution 1:3000), anti-N-Cadherin rabbit mAb (CST, 13116; dilution 1:1000), anti-LAMP2[H4B4] mouse mAb (Abcam, ab25631; dilution 1:1000), anti-LAMP2A rabbit pAb (Abcam, ab18528; dilution 1:1000), anti-αTubulin mouse mAb (Abcam, ab7291; dilution 1:5000), anti-βactin mouse mAb (Sigma, A5316; dilution 1:5000) were used for immunoblot and immunoprecipitation assay.

### Immunofluorescence

For immunofluorescence assay, 1000 cells of HBE, A549 or H1299 cells were cultured on a circle microscope cover glass in a 24-well plate. Twenty-four hours later, the cells were fixed with 4% paraformaldehyde for 10 min, permeabilized with 0.3% Triton-100 for 10 min, and blocked with 2% BSA for 1 h at room temperature. The cells were then incubated with the primary antibodies against CD47 (Abcam, ab3283; dilution 1:100), RAGA (CST, 4357; dilution 1:100), LAMP1 (CST, 9091; dilution 1:250), RAB7 (CST, 9367; dilution 1:250) at 4°C overnight respectively, washed with PBS three times and incubated with fluorescence-conjugated second antibodies for 1 h at room temperature. The cells were washed for additional three times with PBS and co-mounted with DAPI. Images were taken with a confocal microscope. The fluorescence intensity was quantified by using the ImageJ software. The colocalization was analyzed by using manders’ coefficients in ImageJ JaCop plugin. For calculating the PM/intracellular CD47 ratio, the CD47 immunofluorescence signal located at the outmost cell peripheral region that appears as dense lines were determined as the PM CD47 signal and the signal located inside the lines were determined as intracellular CD47. Both PM/intracellular CD47 signal intensity were measured by ImageJ software and the PM/intracellular ratio were calculated. The cells analyzed in this study were unbiased representatives of two independent replicates.

### Isolation of plasma membrane and lysosome

Minute^TM^ Plasma Membrane Protein Isolation and Cell Fraction Kit (Invent, SM-005) was used to isolate the plasma membrane according to the manufacturer’s instructions. Lysosome Enrichment Kit for Tissues and Cultured Cell (Thermo Scientific, 89839) was used to enrich lysosomes according to the manufacturer’s protocol. The purified plasma membrane and lysosome were lysed in RIPA lysis buffer and analyzed by immunoblot for indicated proteins.

### Flow cytometry

Fluorescence dye-conjugated antibodies for the flow cytometry analysis include PE-conjugated anti-Human CD47(B6H12) antibody (BD, 556046; 5 μl per sample), PE-conjugated anti-mouse CD11b antibody (Biolegend, 101207; 1 μl per sample), APC-conjugated anti-mouse F4/80 antibody (R&D, FAB5580A; 1 μl per sample). About 2–10 × 10^5^ cells were resuspended in 100 μl PBS containing 2% FBS and stained with the indicated fluorescence antibodies for 30 min on ice in dark and the flow cytometry were then performed on LSRFortessaTM cytometer (BD Biosciences) or CytoFLEX cytometer (Beckman). The data were analyzed by using the Flowjo software. To calculate the “Relative MFI”, we measured the mean of fluorescence intensity (MFI) by using flowjo software and then calculated the ratio of MFI of RAGA knockdown cells to MFI of shControl cells or the ratio of MFI of RAGA overexpression cells to MFI of pBabe cells. The ratios were indicated as “Relative MFI”.

### In vitro phagocytosis assay

The primary bone marrow-derived macrophages were generated from mouse bone marrow cells according to a modified protocol^54,55^. Briefly, mouse bone marrow cells were isolated from both mouse femurs and tibias and cultured in RPMI 1640 (Gibco) complete medium supplied with 30% supernatant of L929 cells. The cells were cultured for 3 days and additional 30% supernatant of L929 cell was added. Differentiated macrophages were obtained at day 7 and dissociated from the culture dish with StemPro Accutase Cell Dissociation Reagent (A1110501)(Gibco). The macrophages were then used for in vitro phagocytosis assay, 1 × 10^6^ macrophage cells were resuspended with 1 × 10^6^ tumor cells (GFP-labeled) in 100 μl RPMI 1640 and incubated for 2 h at 37 °C. About 15 μg/ml anti-human CD47 antibody (B6H12) (Santa Cruz, sc-12730) was added to the CD47 blockade groups. The cells were then collected and stained with APC-conjugated mouse F4/80 antibody (R&D, FAB5580A; 2 μl per sample) for 30 min to label macrophages, followed by flow cytometry analysis.

### Mouse tumor xenograft and in vivo phagocytosis assay

The 6–8-week age female Balb/c nude mice were randomly divided into indicated groups. About 5 × 10^6^ GFP-labeled A549 control or RAGA knockdown cells were resuspended in 1×HBSS without calcium and magnesium and injected into the nude mouse subcutaneously. For the CD47 antibody treatment, the mice were injected intraperitoneally with 200 μg/kg IGG and CD47 antibody every other day after 7 days of tumor injection when the tumor is palpable. Both short (W) and long (L) diameters of tumor xenografts were measured, and the volumes (V) of xenografts were calculated according to the formula V = L × W^2^ × 0.5. At the end of the mouse experiment, the tumor xenografts were isolated and weighed. For in vivo phagocytosis analysis, the tumor tissues were digested and single-cell suspensions were prepared. About 1 × 10^6^ cells were stained with PE-conjugated mouse CD11b antibody (Biolegend, 101207; 1 μl per sample) and APC-conjugated mouse F4/80 antibody (R&D, FAB5580A; 1 μl per sample) followed by flow cytometry. All mouse experiments were in accordance with ethical standards approved by responsible committees of Guangdong Provincial People’s Hospital.

### Human-patient material analysis

The human lung adenocarcinoma analysis was performed on a commercial tissue chip containing 94 adenocarcinoma tissues. Eighty-four patients with survival information available were used for follow-up analysis in this study. Immunohistochemistry staining of both CD47 (Proteintech, 20305-1-AP; dilution 1:1000) and RAGA (Abcam, ab91062; dilution 1:500) were performed on the tissue chip in Shanghai OUTDO Biotech. The immunostaining expression value of CD47 and RAGA was quantified by using ImageJ software, determined by integrated optical density (IOD) value/area. Informed consent was obtained from all participating patients. The study protocol and informed consent are in compliance with all relevant ethical regulations approved by responsible committees in Shanghai OUTDO Biotech.

### Statistics and reproducibility

Statistical analysis was performed using GraphPad Prism software. Patient survival data were statistically analyzed by the log-rank (Mantel–Cox) test. The other data were statistically analyzed by two-sided student’s *t*-test or one-way ANOVA and were presented as mean ± SD. *P* < 0.05 was considered as statistical significance. * indicates *P* < 0.05; ** indicates *P* < 0.01; *** indicates *P* < 0.001; and **** indicates *P* < 0.0001. The sample and replicate size were indicated in the figure legends.

### Reporting summary

Further information on research design is available in the Nature Portfolio Reporting Summary linked to this article.

## Supplementary information

Supplementary Information

Description of Additional Supplementary Files

Supplementary Data 1

Reporting Summary

## Data Availability

All data supporting this study are available from the corresponding authors on reasonable request. The unprocessed blot images are included in Supplementary Fig. 7. The source data behind the graphs in the paper are included in Supplementary Data 1.
