# Supplementary material for: RAGA prevents tumor immune evasion of LUAD by promoting CD47 lysosome degradation
Source: Commun Biol. 2023 Feb 23;6:211. doi: 10.1038/s42003-023-04581-z (PMC9950044; doi:10.1038/s42003-023-04581-z)
Supplement: Supplementary file 1 — Supplementary Information [file 42003_2023_4581_MOESM1_ESM.pdf]

Supplementary Materials for

RAGA prevents tumor immune evasion of LUAD by promoting CD47 lysosome degradation

Lian Zhang, Jing Yu, Mingyue Zheng, Hui Zhen, Qingqiang Xie, Chundong Zhang, Zhongjun Zhou*, Guoxiang Jin*

*Corresponding author: Guoxiang Jin (gxjinking@163.com) and Zhongjun Zhou (zhongjun@hku.hk).

This file includes:

Supplementary Fig. 1 to 7

Supplementary Table 1

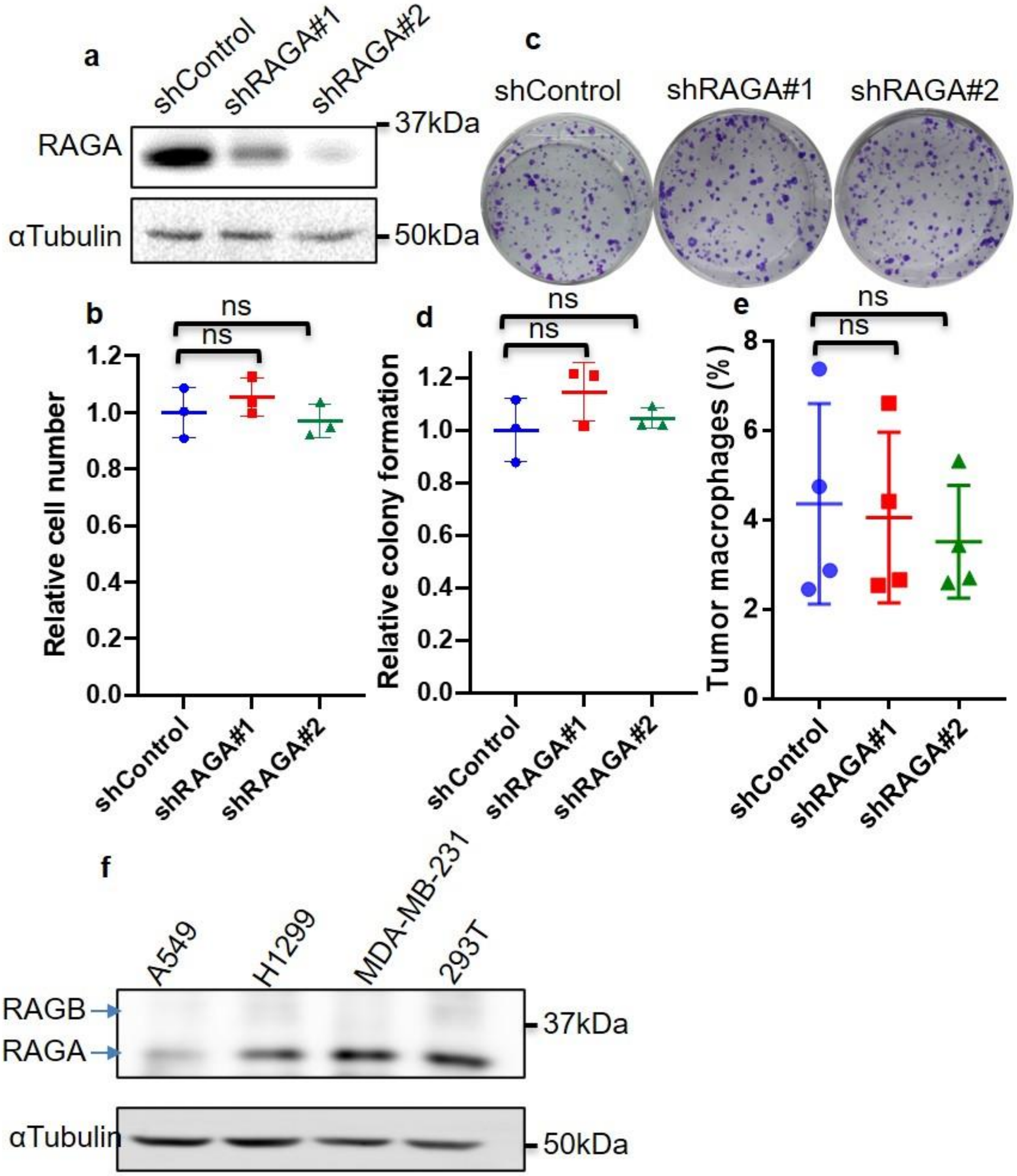

Supplementary Fig. 1 The role of RAGA in lung cancer cell proliferation *in vitro*. a Immunoblot analysis of indicated proteins in control and RAGA knockdown H1299 cells. **b** Relative cell number of control and RAGA knockdown H1299 cells (n=3 per group). **c** Representative images of control and RAGA knockdown H1299 colonies. **d** Relative colony number of control and RAGA knockdown H1299 cells (n=3 per group). **e** The percentage of tumor macrophage infiltration (n=4 per gruop) **f** Immunoblot analysis of RAGA and RAGB in A549, H1299, MDA-MB-231 and 293T cells. Data are from two

independent experiments (**a**, **c**, **d**, **e**) or three independent experiments (**b**). Statistical data are presented as mean±SD. *P*<0.05 is considered as statistical significance. ns indicates not significant.

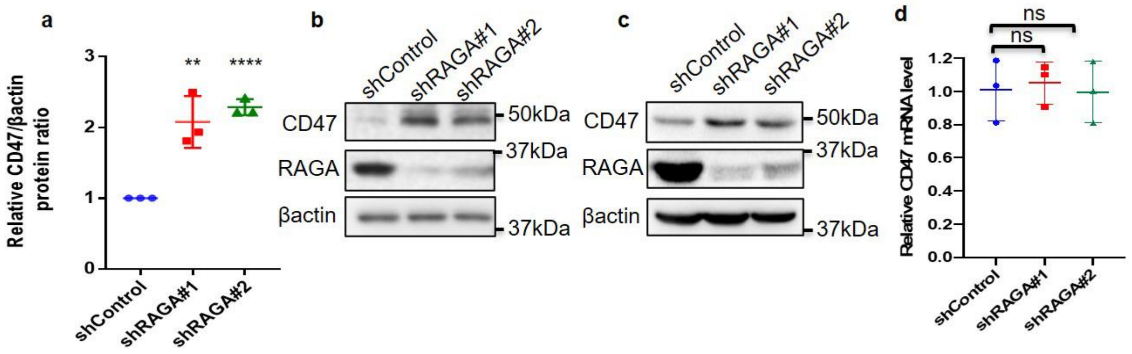

Supplementary Fig. 2 RAGA inhibits CD47 protein level. a Quantification of CD47/βactin protein ratio in Fig.2a. **b.** Immunoblot analysis of indicated proteins in control and RAGA knockdown MDA-MB-231 cells. **c** Immunoblot analysis of indicated proteins in control and RAGA knockdown H1299 cells. **d** qRT-PCR analysis of *CD47* mRNA in control and RAGA knockdown A549 cells. Statistical data are presented as mean±SD. *P*<0.05 is considered as statistical significance. ns indicates not significant.

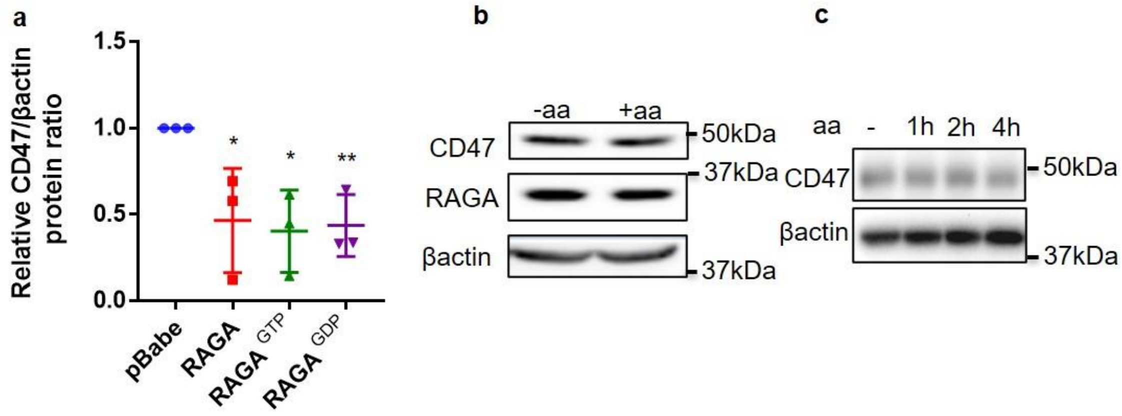

Supplementary Fig. 3 CD47 protein level independent of amino acid stimulation. a Quantification of CD47/βactin protein ratio in Fig.3a. **b-c** A549 cells were cultured in RPMI 1640 medium without amino acids for 2 hours and then the medium was replaced with complete RPMI 1640 (+aa) or RPMI 1640 without amino acids (-aa) for additional 2 hours (**b**) or (1, 2, 4) hours (**c**). Immunoblot analysis was performed for the indicated proteins. Statistical data are presented as mean±SD. *P*<0.05 is considered as statistical significance.

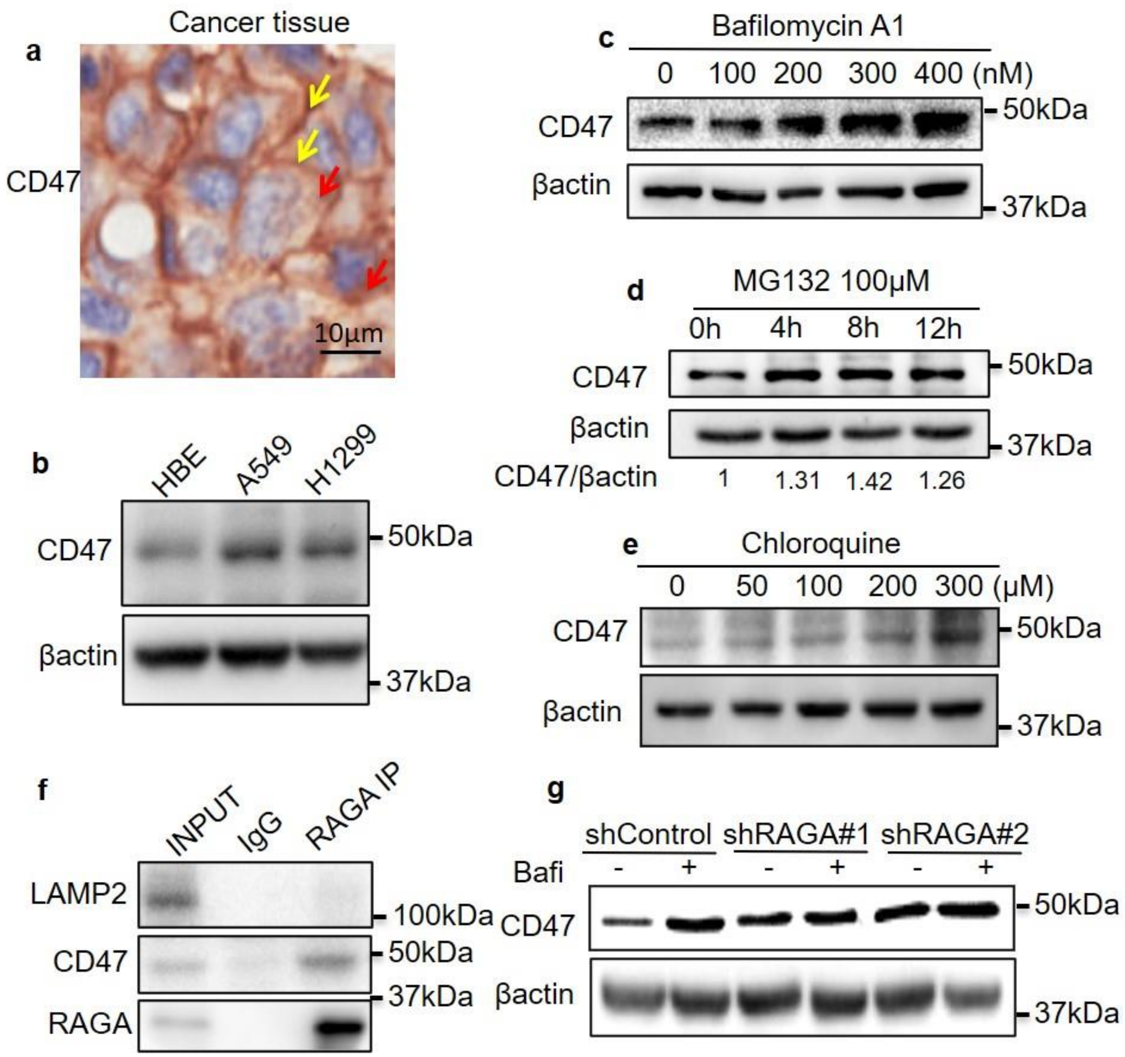

Supplementary Fig. 4 RAGA promotes CD47 lysosome degradation. a CD47 immunohistochemistry in lung adenocarcinoma tissue of human patient. Yellow arrow indicates cell surface CD47 staining. Red arrow indicates intracellular CD47 staining. **b** Immunoblot analysis of CD47 in HBE, A549 and H1299 cells. **c** Immunoblot analysis of the indicated proteins in H1299 cells treated with different dosage of bafilomycin A1. **d** Immunoblot analysis of the indicated proteins in A549 cells treated with 100μM MG132. h indicates hours. **e** Immunoblot analysis of the indicated proteins in H1299 cells treated with different dosage of chloroquine. **f** Immunoblot analysis of indicated proteins assessed after RAGA immunoprecipitation. **g** Immunoblot analysis of indicated proteins in control and RAGA knockdown H1299 cells treated with or without bafilomycin A1. Bafi indicates bafilomycin A1.

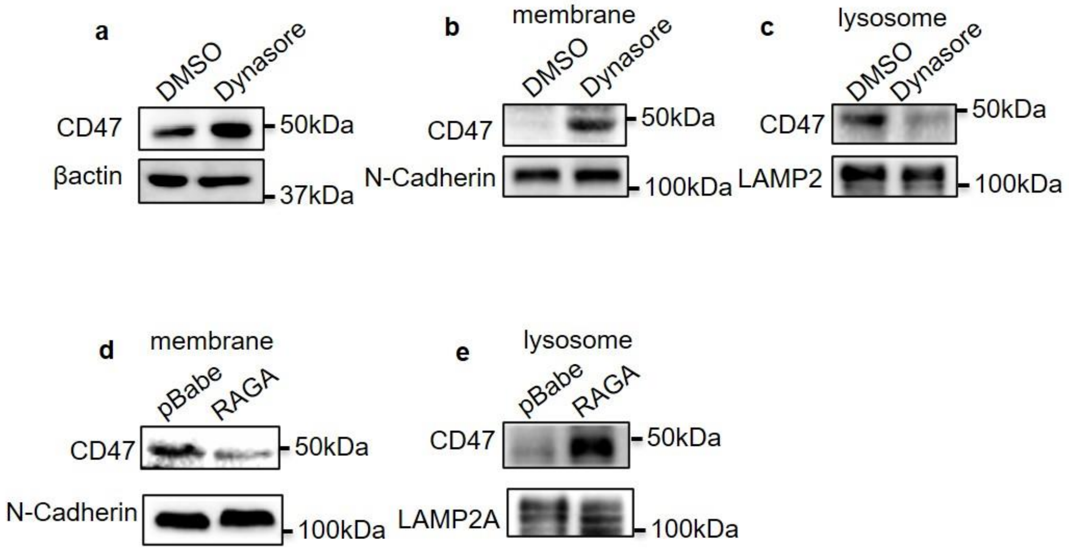

Supplementary Fig. 5 RAGA promotes CD47 lysosome recruitment. a Immunoblot analysis of indicated proteins in A549 cells with or without dynasore treatment (80 μM). **b** Immunoblot analysis of indicated proteins in cell plasma membrane fraction with or without dynasore treatment (80 μM). **c** Immunoblot analysis of indicated proteins in cell lysosome with or without dynasore treatment (80 μM). **d** Immunoblot analysis of indicated proteins in the isolated plasma membrane fraction of empty vector and RAGA overexpressed A549 cells. **e** Immunoblot analysis of indicated proteins in the isolated lysosome fraction of empty vector and RAGA overexpressed A549 cells. Data are from two independent experiments.

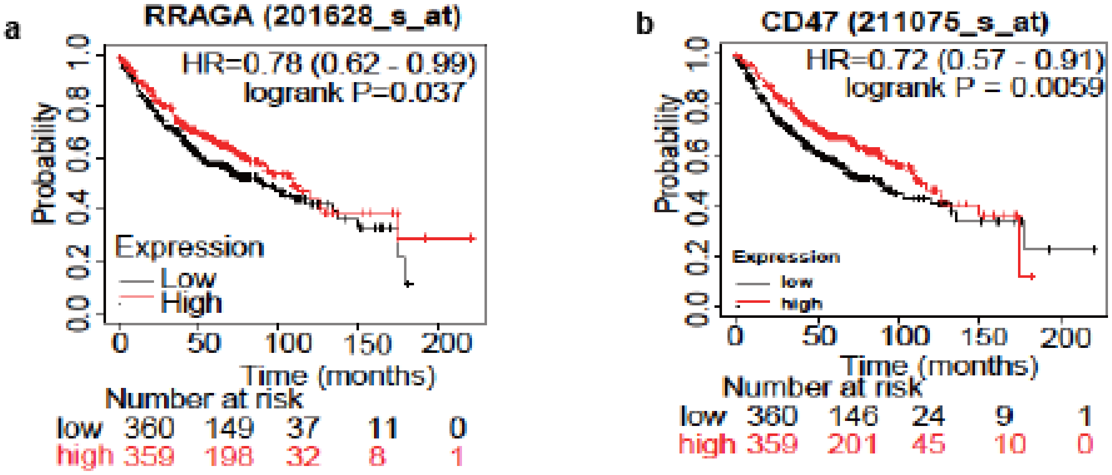

Supplementary Fig. 6 The association of *RAGA/CD47* mRNA level with lung adenocarcinoma patient survival. a Overall survival analysis of GEO lung

adenocarcinoma patient database with low and high *RAGA (RRAGA)* mRNA expression in kmplot.com website. *P*=0.037. **b** Overall survival analysis of GEO lung adenocarcinoma patient database with low and high *CD47* mRNA expression in kmplot.com website. *P*=0.0059. *P*<0.05 is considered as statistical significance.

Supplementary Fig. 7 Unprocessed Blots

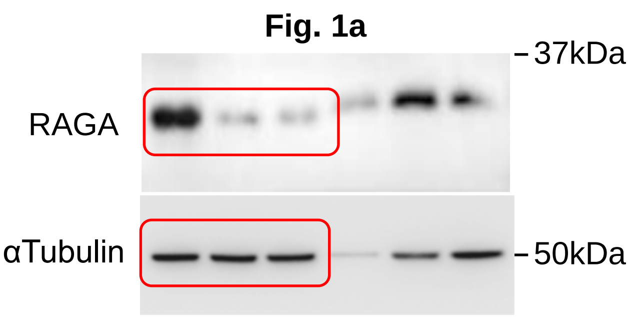

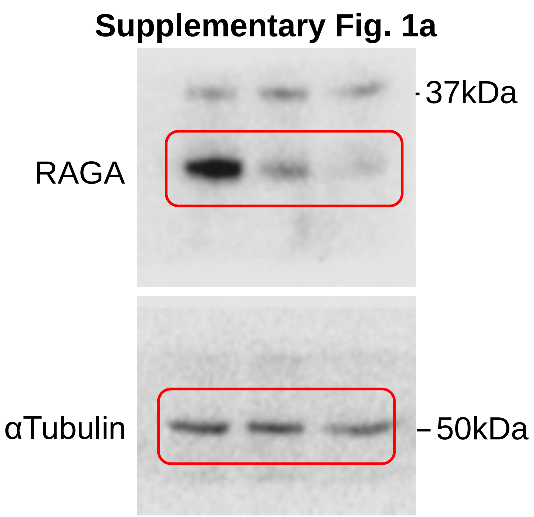

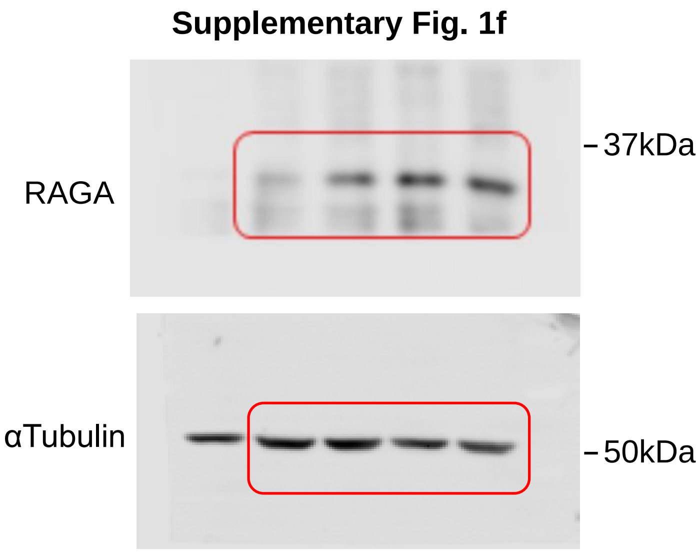

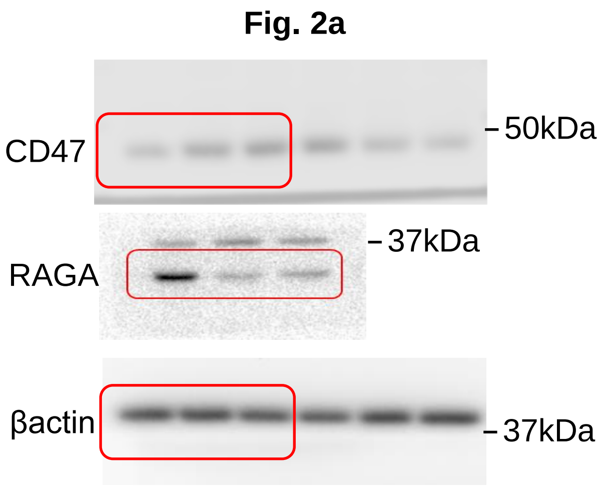

Supplementary Fig. 2b

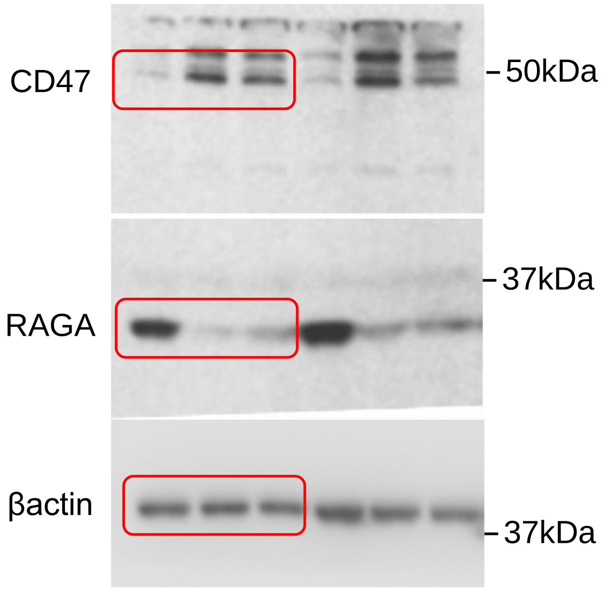

Supplementary Fig. 2c

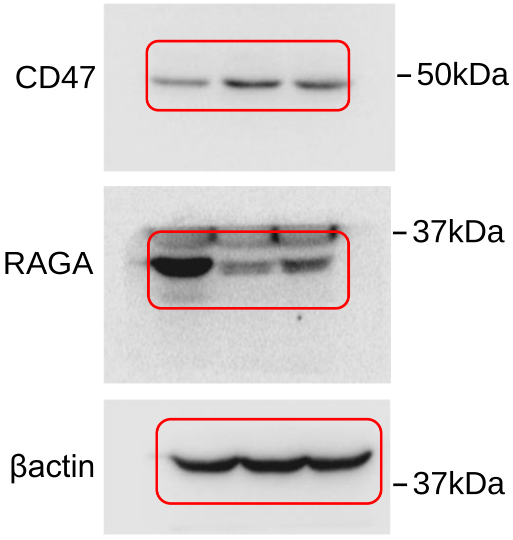

Fig. 3a

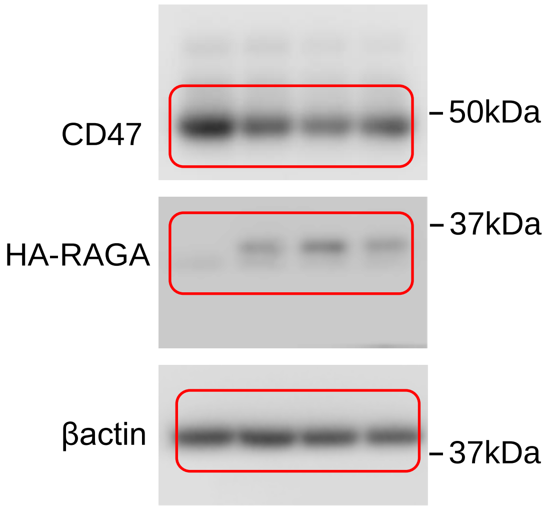

Supplementary Fig. 3b

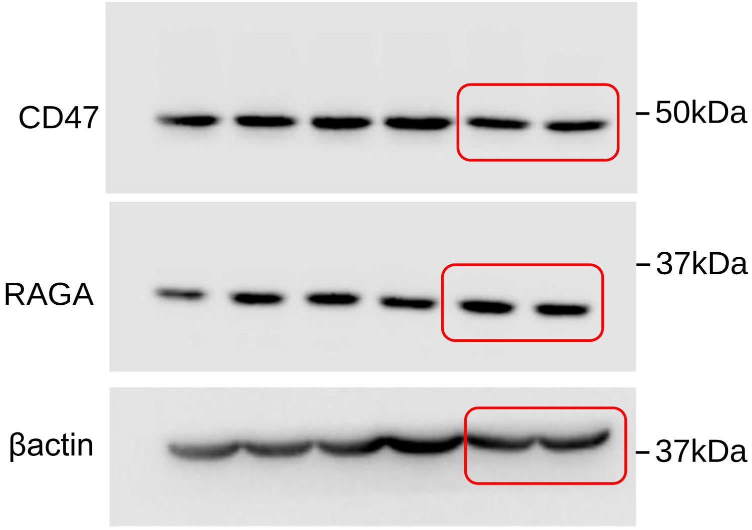

Supplementary Fig. 3c

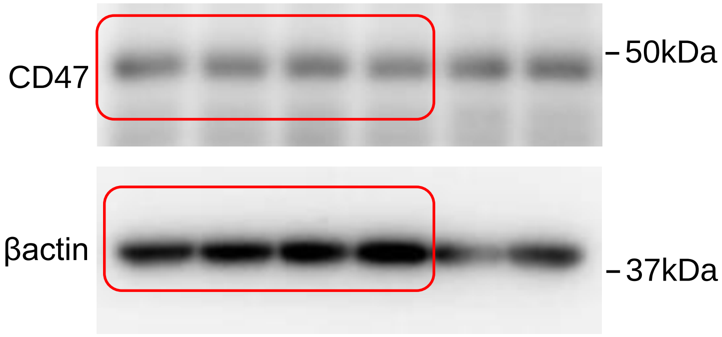

Fig. 4d

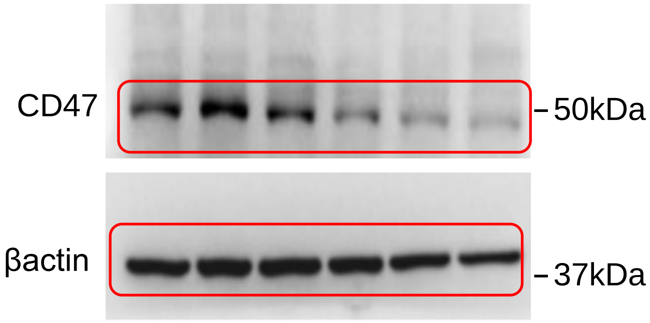

Fig. 4e

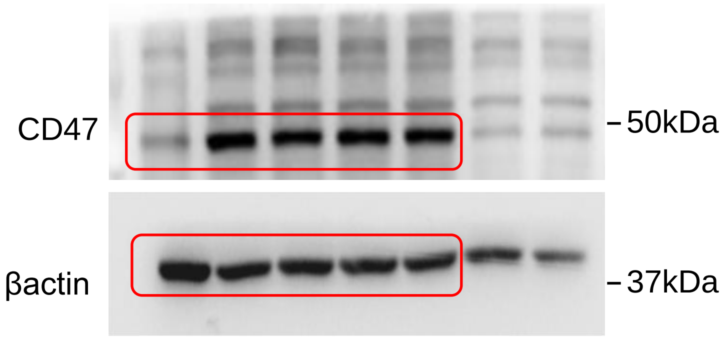

Fig. 4f

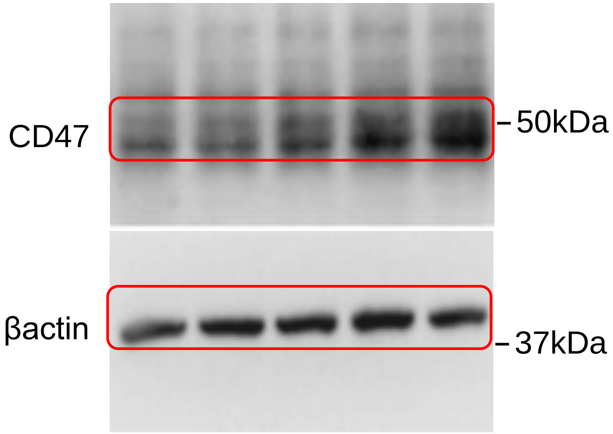

Fig. 4h

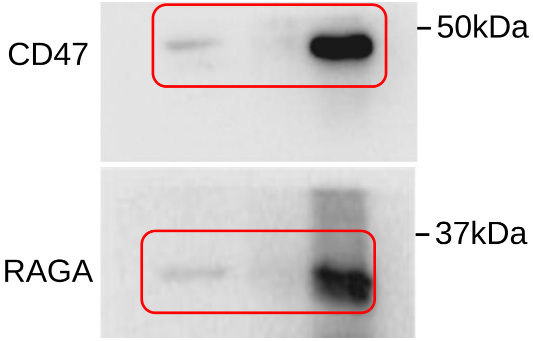

Fig. 4i

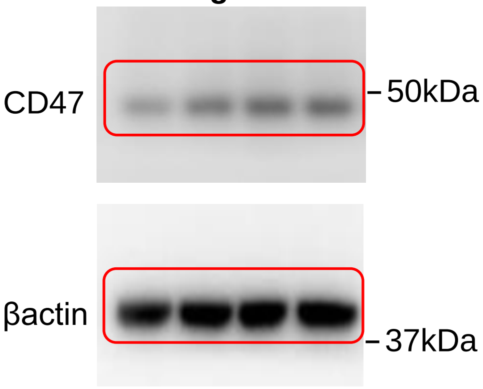

Supplemental Fig. 4b

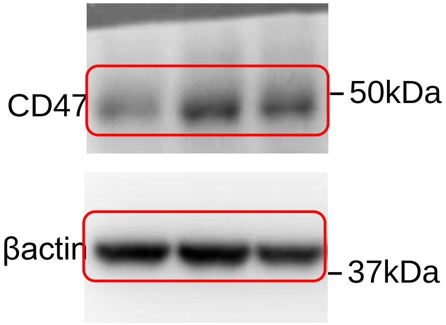

Supplemental Fig. 4c

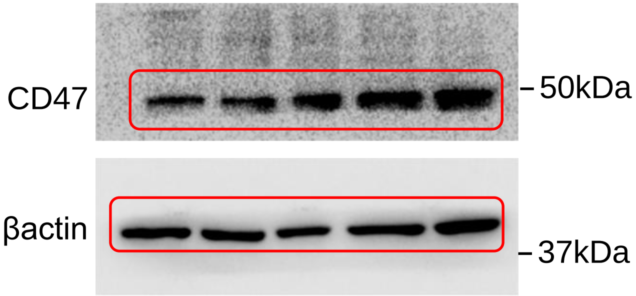

Supplemental Fig. 4d

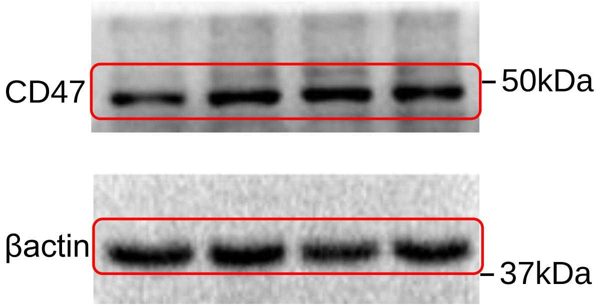

Supplemental Fig. 4e

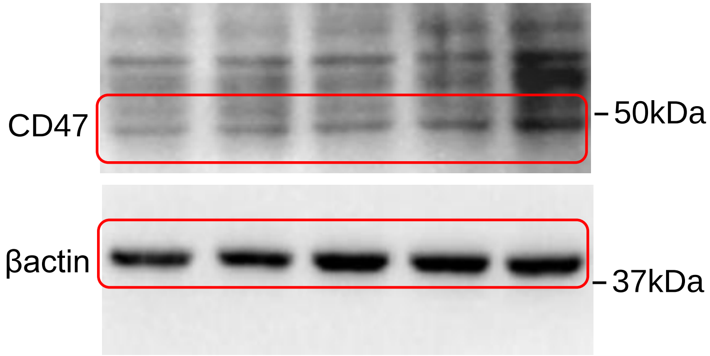

Supplemental Fig. 4f

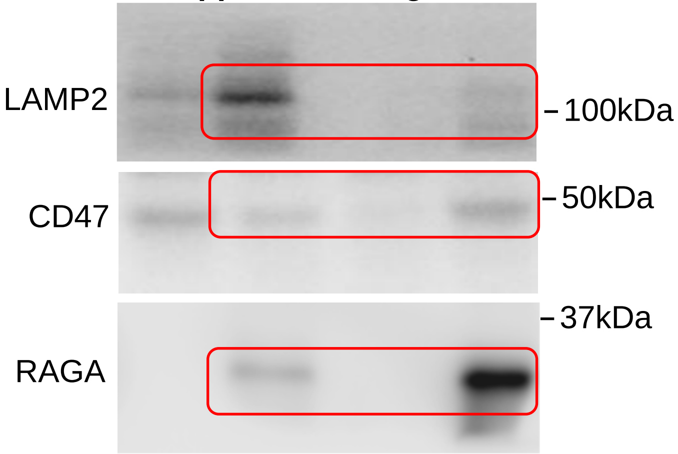

Supplemental Fig. 4g

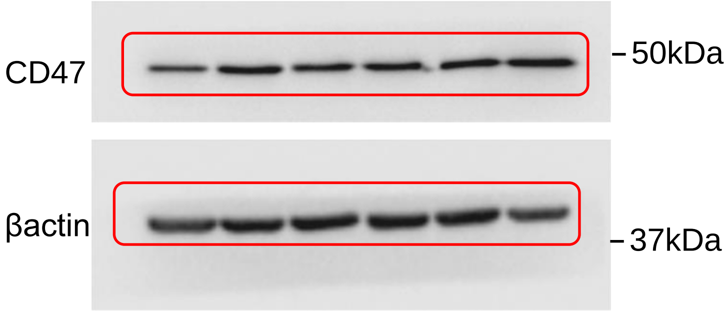

Fig. 5d

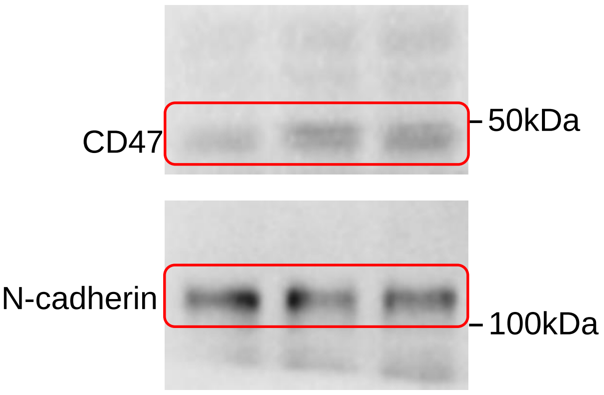

Fig. 5e

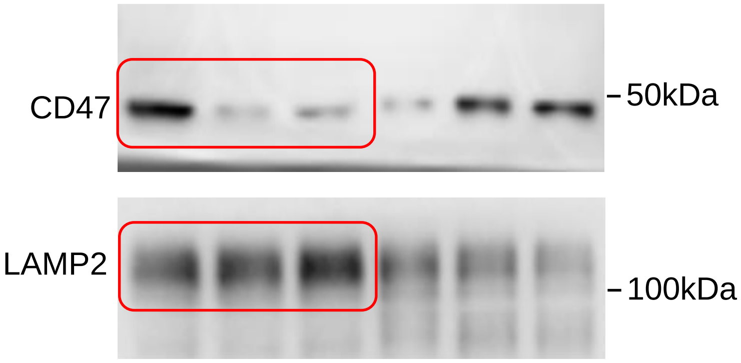

Fig. 5i

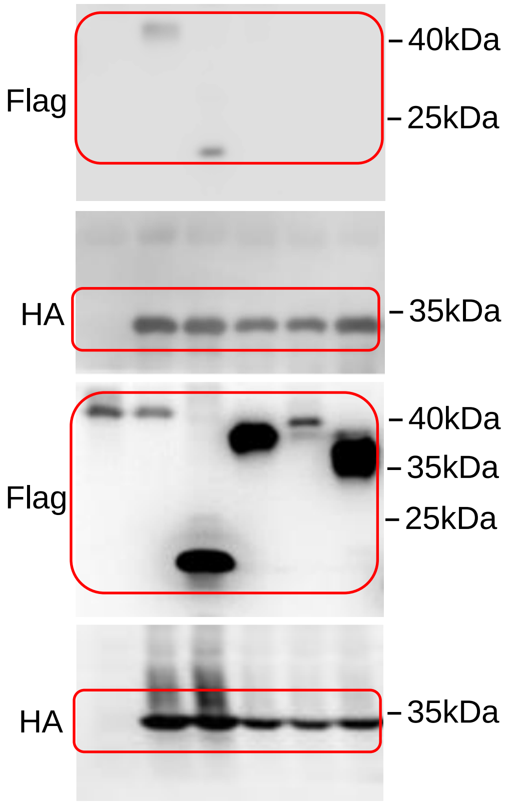

Supplemental Fig. 5a

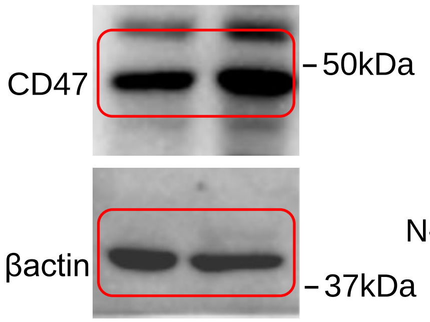

Supplemental Fig. 5b

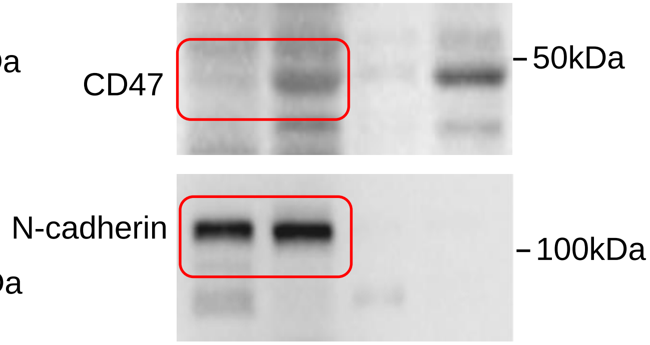

Supplemental Fig. 5c

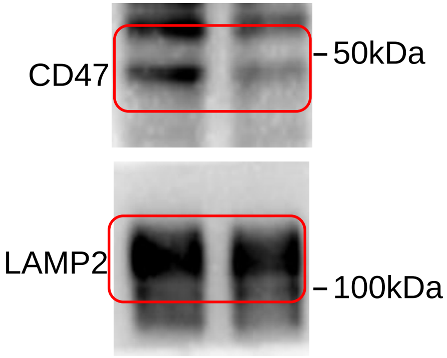

Supplemental Fig. 5d

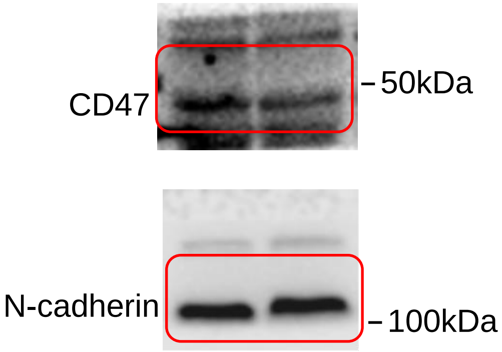

Supplemental Fig. 5e

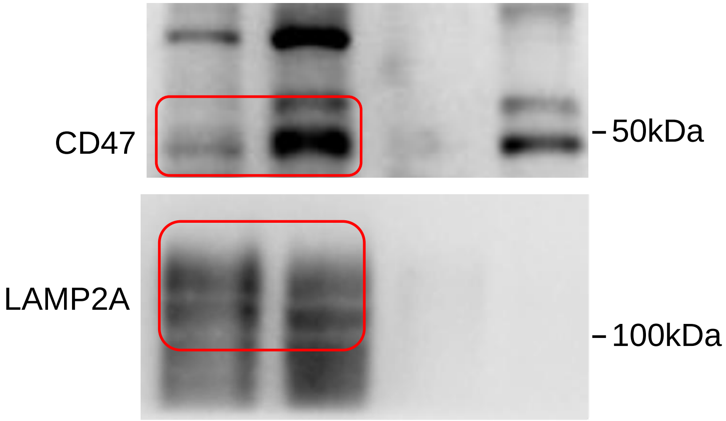

Supplemental Table 1 . List of lung adenocarcinoma patients

Patient No.	Alive-0, Dead-1	Live months	Gender	Age	T	N	M	Stage
01	0	121	Female	84	T1a	N0	M0	1A
02	1	78	Female	72	T1b	N0	M0	1A
03	1	38	Female	59	T3	N1	M0	3A
04	1	49	Female	66	T2a	Nx	M0	2-3
05	0	116	Male	49	T1b	N0	M0	1A
06	0	113	Female	53	T1b	N0	M0	1A
07	1	33	Male	74	T2a	Nx	M0	2-3
08	1	21	Male	74	T2a	N0	M0	1B
09	1	39	Male	58	T2a	N0	M0	1B
10	1	34	Male	30	T1b	N0	M0	1A
11	1	39	Male	67	T2b	Nx	M0	2-3
12	1	15	Female	64	T2a	N0	M0	1B
13	1	52	Female	52	T2a	Nx	M0	2-3
14	1	62	Male	60	T2a	N0	M0	1B
15	1	33	Male	47	T3	N1	M0	3A
16	1	14	Male	65	T3	N2	M0	3A
17	1	49	Female	58	T2a	N0	M1b	4
18	1	13	Female	67	T2a	N1	M0	2A
19	0	92	Female	50	T1b	N0	M0	1A
20	1	57	Female	60	T3	N1	M0	3A
21	0	91	Female	68	T1b	N0	M0	1A
22	0	91	Male	55	T2a	N0	M0	1B
23	1	15	Female	76	T1b	N0	M0	1A
24	1	17	Male	49	T2a	N2	M0	3A
25	1	33	Male	73	T2b	N1	M0	2B
26	1	14	Female	58	T4	N1	M0	3A
27	1	59	Male	75	T2a	N1	M0	2A
28	1	48	Female	69	T2a	Nx	M0	2-3
29	1	27	Male	75	T4	N0	M0	3A
30	1	44	Female	52	T3	N3	M0	3B
31	0	87	Male	55	T2a	N0	M0	1B
32	1	25	Male	65	T2a	N1	M0	2A
33	1	13	Male	42	T1b	Nx	M0	2-3
34	1	29	Female	51	T1a	N2	M0	3A
35	1	16	Male	53	T2a	N2	M0	3A
36	1	6	Male	66	T2a	N1	M0	2A
37	1	40	Female	57	T2a	Nx	M0	2-3

38	1	57	Female	51	T2a	Nx	M0	2-3
39	1	33	Male	71	T3	N1	M0	3A
40	1	40	Female	60	T3	N3	M0	3B
41	1	15	Male	61	T2b	N3	M0	3B
42	1	35	Female	58	T3	N0	M0	2B
43	0	79	Male	60	T3	N1	M0	3A
44	1	25	Male	63	T3	N2	M0	3A
45	1	49	Male	63	T2a	N1	M0	2A
46	1	39	Male	61	T2a	N0	M0	1B
47	1	58	Female	81	T2a	N1	M0	2A
48	1	7	Male	61	T2b	N0	M0	2A
49	1	24	Male	84	T2b	N0	M0	2A
50	0	75	Male	65	T2a	N2	M0	3A
51	1	52	Male	53	T2a	N0	M0	1B
52	1	1	Male	74	T1a	——	M0	——
53	0	74	Male	64	T2a	N1	M0	2A
54	1	54	Female	73	T4	N2	M0	3B
55	1	14	Male	52	T2a	N2	M0	3A
56	1	3	Male	44	T3	Nx	M0	3
57	1	12	Male	55	T2b	N2	M0	3A
58	1	15	Female	50	T1a	N0	M0	1A
59	1	39	Male	78	T1b	N0	M0	1A
60	1	2	Female	60	T2b	N0	M0	2A
61	1	29	Female	54	T3	N1	M0	3A
62	0	68	Female	48	T3	N0	M0	2B
63	1	2	Male	59	T2a	N3	M0	3B
64	1	50	Male	48	T2a	N2	M0	3A
65	1	25	Male	59	T2a	Nx	M0	2-3
66	0	68	Male	58	T2a	N0	M0	1B
67	1	15	Female	56	T4	N2	M0	3B
68	0	67	Female	53	T2a	N0	M0	1B
69	1	10	Female	62	T1b	Nx	M0	2-3
70	1	30	Male	72	T2a	N2	M0	3A
71	0	65	Male	61	T2b	N0	M0	2A
72	1	55	Female	65	T2a	N0	M0	1B
73	0	64	Female	67	T1b	Nx	M0	2-3
74	0	64	Male	65	T3	N0	M0	2B
75	1	29	Female	77	T3	N3	M0	3B
76	0	62	Female	66	T2a	N0	M0	1B
77	1	3	Female	57	T4	N2	M0	3B
78	1	56	Male	74	T2a	N0	M0	1B

79	0	84	Female	71	T1b	N0	M0	1A
80	0	80	Female	58	T3	N0	M0	2B
81	1	72	Female	62	T1a	Nx	M0	2-3
82	1	12	Female	73	T2b	Nx	M0	2-3
83	1	2	Male	54	T4	N2	M0	3B
84	1	8	Male	65	T3	N2	M0	3A
