# Supplementary material for: RAGA prevents tumor immune evasion of LUAD by promoting CD47 lysosome degradation
Source: Commun Biol. 2023 Feb 23;6:211. doi: 10.1038/s42003-023-04581-z (PMC9950044; doi:10.1038/s42003-023-04581-z)
Supplement: Supplementary file 4 — Reporting Summary [file 42003_2023_4581_MOESM4_ESM.pdf]

nature portfolio

Corresponding author(s): Guoxiang Jin

Last updated by author(s): 01/29/2023

Reporting Summary

Nature Portfolio wishes to improve the reproducibility of the work that we publish. This form provides structure for consistency and transparency in reporting. For further information on Nature Portfolio policies, see our Editorial Policies and the Editorial Policy Checklist.

Statistics

For all statistical analyses, confirm that the following items are present in the figure legend, table legend, main text, or Methods section.

n/a	Confirmed	
☐	☒	The exact sample size (n) for each experimental group/condition, given as a discrete number and unit of measurement
☐	☒	A statement on whether measurements were taken from distinct samples or whether the same sample was measured repeatedly
☐	☒	The statistical test(s) used AND whether they are one- or two-sided<br>*Only common tests should be described solely by name; describe more complex techniques in the Methods section.*
☒	☐	A description of all covariates tested
☒	☐	A description of any assumptions or corrections, such as tests of normality and adjustment for multiple comparisons
☐	☒	A full description of the statistical parameters including central tendency (e.g. means) or other basic estimates (e.g. regression coefficient) AND variation (e.g. standard deviation) or associated estimates of uncertainty (e.g. confidence intervals)
☒	☐	For null hypothesis testing, the test statistic (e.g. F, t, r) with confidence intervals, effect sizes, degrees of freedom and P value noted<br>*Give P values as exact values whenever suitable.*
☒	☐	For Bayesian analysis, information on the choice of priors and Markov chain Monte Carlo settings
☒	☐	For hierarchical and complex designs, identification of the appropriate level for tests and full reporting of outcomes
☒	☐	Estimates of effect sizes (e.g. Cohen's d, Pearson's r), indicating how they were calculated

Our web collection on statistics for biologists contains articles on many of the points above.

Software and code

Policy information about availability of computer code

Data collection	NA
Data analysis	NA

For manuscripts utilizing custom algorithms or software that are central to the research but not yet described in published literature, software must be made available to editors and reviewers. We strongly encourage code deposition in a community repository (e.g. GitHub). See the Nature Portfolio guidelines for submitting code & software for further information.

Data

Policy information about availability of data

All manuscripts must include a data availability statement. This statement should provide the following information, where applicable:

- Accession codes, unique identifiers, or web links for publicly available datasets
- A description of any restrictions on data availability
- For clinical datasets or third party data, please ensure that the statement adheres to our policy

All relevant data are available from the corresponding author upon reasonable request.

Human research participants

Policy information about studies involving human research participants and Sex and Gender in Research.

Reporting on sex and gender	Sen and gender were not considered in this study
Population characteristics	All population characteristics were provided in Supplementary Table 1
Recruitment	All patient information were provided by Shanghai OUTDO Biotech
Ethics oversight	The study protocol is in compliance with all relevant ethical regulations approved by responsible committees of Shanghai OUTDO Biotech

Note that full information on the approval of the study protocol must also be provided in the manuscript.

Field-specific reporting

Please select the one below that is the best fit for your research. If you are not sure, read the appropriate sections before making your selection.

☒ Life sciences ☐ Behavioural & social sciences ☐ Ecological, evolutionary & environmental sciences

For a reference copy of the document with all sections, see nature.com/documents/nr-reporting-summary-flat.pdf

Life sciences study design

All studies must disclose on these points even when the disclosure is negative.

Sample size	The sample sizes were chosen following previous publications and the requirement of statistical analysis. No sample size calculations were performed.
Data exclusions	No data were excluded.
Replication	All main data were from at least two independent experiments.
Randomization	The mice used for xenograft experiments were randomly divided into subgroups.
Blinding	Investigators were blinded to group allocation of xenograft experiments and immunostaing scoring of tissue samples

Reporting for specific materials, systems and methods

We require information from authors about some types of materials, experimental systems and methods used in many studies. Here, indicate whether each material, system or method listed is relevant to your study. If you are not sure if a list item applies to your research, read the appropriate section before selecting a response.

Materials & experimental systems

n/a	Involved in the study	
☐	☒	Antibodies
☐	☒	Eukaryotic cell lines
☒	☐	Palaeontology and archaeology
☐	☒	Animals and other organisms
☒	☐	Clinical data
☒	☐	Dual use research of concern

Methods

n/a	Involved in the study	
☒	☐	ChIP-seq
☐	☒	Flow cytometry
☒	☐	MRI-based neuroimaging

Antibodies

Antibodies used	anti-RAGA(D8B5) rabbit mAb (CST, 4357), anti-CD47 rabbit mAb (Abcam, ab175388), anti-CD47 mouse mAb (Abcam, ab3283), anti-CD47 (Proteintech, 20305-1-AP), anti-RAGA (Abcam, ab91062), anti-HA(HA-7) mouse mAb (Sigma, H9658), anti-N-Cadherin(D4R1H) rabbit mAb (CST, 13116), anti-LAMP2[H4B4] mouse mAb (Abcam, ab25631), anti-βactin(AC-74) mouse mAb (Sigma, A5316) ,anti-LAMP1(D2D11) rabbint mAb (CST, 9091), anti-Rab7 (D95F2) XP® Rabbit mAb (CST, 9367),anti-Human CD47(B6H12) antibody (BD, 556046), PE-conjugated anti-mouse CD11b(M1/70) antibody (Biolegend, 101207), APC-conjugated anti-mouse F4/80 antibody (R&D, FAB5580A)

Validation	anti-RAGA(D8B5) rabbit mAb (CST, 4357), validation: https://www.cellsignal.com/products/primary-antibodies/raga-d8b5-rabbit-mab/4357?site-search-type=Products&N=4294956287&Ntt=raga&fromPage=plp<br>anti-CD47 rabbit mAb (Abcam, ab175388), validation: https://www.abcam.com/cd47-antibody-ab175388.html<br>anti-CD47 mouse mAb (Abcam, ab3283), validation: https://www.abcam.com/cd47-antibody-b6h122-ab3283.html<br>anti-CD47 (Proteintech, 20305-1-AP),validation:https://www.ptglab.com/products/CD47-Antibody-20305-1-AP.htm<br>anti-RAGA (Abcam, ab91062), validation: https://www.abcam.com/rraga--rragb-antibody-ab91062.html<br>anti-HA(HA-7) mouse mAb (Sigma, H9658), validation: https://www.sigmaaldrich.cn/CN/en/product/sigma/h9658?context=product<br>anti-N-Cadherin(D4R1H) rabbit mAb (CST, 13116), validation: https://www.cellsignal.com/products/primary-antibodies/n-cadherin-d4r1h-xp-rabbit-mab/13116<br>anti-LAMP2[H4B4] mouse mAb (Abcam, ab25631), validation: https://www.abcam.com/lamp2-antibody-h4b4-lysosome-marker-ab25631.html<br>anti-βactin(AC-74) mouse mAb (Sigma, A5316) ,validation: https://www.sigmaaldrich.cn/CN/en/product/sigma/a5316?context=product<br>anti-LAMP1(D2D11) rabbint mAb (CST, 9091), validation:https://www.cellsignal.com/products/primary-antibodies/lamp1-d2d11-xp-rabbit-mab/9091<br>anti-Rab7 (D95F2) XP® Rabbit mAb (CST, 9367),validation: https://www.cellsignal.com/products/primary-antibodies/rab7-d95f2-xp-rabbit-mab/9367<br>anti-Human CD47(B6H12) antibody (BD, 556046), validation: https://www.bdbiosciences.com/en-us/products/reagents/flow-cytometry-reagents/research-reagents/single-color-antibodies-ruo/pe-mouse-anti-human-cd47.556046<br>PE-conjugated anti-mouse CD11b(M1/70) antibody (Biolegend, 101207), validation: https://www.biolegend.com/en-us/products/pe-anti-mouse-human-cd11b-antibody-349?GroupID=BLG10552<br>APC-conjugated anti-mouse F4/80 antibody (R&D, FAB5580A),validation: https://www.rndsystems.com/cn/products/mouse-f4-80-apc-conjugated-antibody-521204_fab5580a

Eukaryotic cell lines

Policy information about cell lines and Sex and Gender in Research

Cell line source(s)	The primary mouse macrophages were generated for mouse bone marrow cells as described in manuscript. A549, H1299 and HEK293T were originally from ATCC.
Authentication	The cell lines we used have been authenticated.
Mycoplasma contamination	All cell lines were negative for mycoplasma contamination.
Commonly misidentified lines<br>(See ICLAC register)	No misidentified lines

Animals and other research organisms

Policy information about studies involving animals; ARRIVE guidelines recommended for reporting animal research, and Sex and Gender in Research

Laboratory animals	The 6-8 week age female Balb/c nude mice were used for xenograft experiments in this study.
Wild animals	No wild animals were used in this study.
Reporting on sex	All female mice were used in this study
Field-collected samples	No field-collected samples were used in this study.
Ethics oversight	All mouse experiments were in accordance with ethical standards approved by responsible committees of Guangdong Provincial People's Hospital.

Note that full information on the approval of the study protocol must also be provided in the manuscript.

Flow Cytometry

Plots

Confirm that:

☒ The axis labels state the marker and fluorochrome used (e.g. CD4-FITC).

☒ The axis scales are clearly visible. Include numbers along axes only for bottom left plot of group (a 'group' is an analysis of identical markers).

☒ All plots are contour plots with outliers or pseudocolor plots.

☒ A numerical value for number of cells or percentage (with statistics) is provided.

Methodology

Sample preparation	For in vitro cultured cells, 2-10×105 cells were resuspended in 100µl PBS containing 2% FBS and stained with the indicated fluorescence antibodies for 30 minutes on ice in dark and the flow cytometry were then performed on LSRFortessaTM cytometer (BD Biosciences) or CytoFLEX cytometer (Beckman). For cells form tumor tissues, the tissues were digested and single cell suspensions were prepared. The cells were stained with PE-conjugated mouse CD11b antibody (Biolegend, 101207) and APC-conjugated mouse F4/80 antibody (R&D, FAB5580A) followed by flow cytometry.
Instrument	LSRFortessaTM cytometer or CytoFLEX cytometer (Beckman)
Software	BD analysis softwares, Flowjo
Cell population abundance	The cell populations were sufficient for analysis
Gating strategy	The gating of positive populations were included in the main figures

☐ Tick this box to confirm that a figure exemplifying the gating strategy is provided in the Supplementary Information.
